# Developmental changes in collenchyma cell-wall polysaccharides in celery (*Apium graveolens* L*.*) petioles

**DOI:** 10.1186/s12870-019-1648-7

**Published:** 2019-02-19

**Authors:** Da Chen, Laurence D. Melton, Zoran Zujovic, Philip J. Harris

**Affiliations:** 1 School of Chemical Sciences, The University of Auckland, Private Bag 92019, Auckland Mail Centre, Auckland, 1142 New Zealand; 2 School of Biological Sciences, The University of Auckland, Private Bag 92019, Auckland Mail Centre, Auckland, 1142 New Zealand

**Keywords:** *Apium graveolens*, Cell elongation, Cellulose, Immunomicroscopy, Monosaccharide composition, Pectic polysaccharides, Polysaccharide mobilities, Primary walls, Solid-state ^13^C NMR spectroscopy, Xyloglucans

## Abstract

**Background:**

Collenchyma cells occur widely in eudicotyledons and provide mechanical support for growing organs. At maturity, the cells are elongated and have thick, non-lignified walls, which in celery contain cellulose and pectic polysaccharides, together with xyloglucans and heteroxylans and heteromannans. A previous study suggested that at least some of the collenchyma cell wall in celery is laid down after expansion has stopped and is thus secondary. In the present study, we re-examined this. We used chemical analysis and immunomicroscopy to determine changes in the polysaccharide compositions of these walls during development. Additionally, solid-state NMR spectroscopy was used to examine changes in polysaccharide mobilities during development.

**Results:**

We showed the collenchyma walls are deposited only during cell expansion, i.e. they are primary walls. During cell-wall development, analytical and immunomicroscopy studies showed that within the pectic polysaccharides there were no overall changes in the proportions of homogalacturonans, but there was a decrease in their methyl esterification. There was also a decrease in the proportions of the (1 → 5)-α-l-arabinan and (1 → 4)-β-d-galactan side chains of rhamnogalacturonan I. The proportions of cellulose increased, and to a lesser extent those of xyloglucans and heteroxylans. Immunomicroscopy showed the homogalacturonans occurred throughout the walls and were most abundant in the middle lamellae and middle lamella junctions. Although the (1 → 4)-β-d-galactans occurred only in the rest of the walls, some of the (1 → 5)-α-l-arabinans also occurred in the middle lamellae and middle lamella junctions. During development, the location of the xyloglucans changed, being confined to the middle lamellae and middle lamella junctions early on, but later occurred throughout the walls. The location of the heteroxylans also changed, occurring mostly in the outer walls in young cells, but were more widely distributed in mature cells. Solid-state NMR spectroscopy showed that particularly cellulose, but also homogalacturonans, decreased in mobility during development.

**Conclusions:**

Our studies showed that celery collenchyma cell walls are primary and that during their development the polysaccharides undergo dynamic changes. Changes in the mobilities of cellulose and homogalacturonans were consistent with the cell walls becoming stiffer as expansion ceases.

**Electronic supplementary material:**

The online version of this article (10.1186/s12870-019-1648-7) contains supplementary material, which is available to authorized users.

## Background

Collenchyma is a simple plant tissue, consisting of only one cell type. Collenchyma cells are elongated, living cells that occur especially in peripheral positions in leaves and stems of eudicotyledons where they provide mechanical support while they are still growing [1–3]. At maturity, the cell walls are thick and usually non-lignified, with the thickening often unevenly distributed. Depending on the positioning of the wall thickening, several types of collenchyma have been recognized, with the most common type being angular collenchyma, where the thickenings occur mostly at the cell corners [4]. This type of collenchyma is found in the petioles of celery (*Apium graveolens*), where it occurs in sub-epidermal strands and is also associated with vascular bundles [1, 4]. The thickened walls of collenchyma cells are usually described as primary [2]. This is based on an early study of the development of collenchyma cell walls in cow parsnip (*Heracleum sphondylium*), where it was shown that the thick walls are laid down early in development before cell expansion ceases and were described as “thickened primary” walls [5]. In contrast, a later study of collenchyma cell walls in celery petioles reported that wall synthesis and assembly continued after cell growth ceased, suggesting that at least some of the wall is secondary [6].

A detailed polysaccharide composition of collenchyma cell walls has only recently been reported for walls obtained from fully elongated petioles of celery [4]. The composition was typical of primary cell walls of eudicotyledons, with cellulose and pectic polysaccharides (pectins) being the most abundant components, and among the pectins, homogalacturonan being more abundant than rhamnogalacturonan I (RG-I) and rhamnogalacturonan II (RG-II). Small amounts of xyloglucans and even smaller amounts of heteroxylans and heteromannans were also found. The composition is similar to that of parenchyma cells in celery petioles, although the collenchyma walls contain somewhat higher proportions of xyloglucans and lower proportions of (1 → 5)-α-l-arabinan and (1 → 4)-β-d-galactan side chains of RG-I [4, 7, 8]. In addition to wet chemical analytical methods, solid-state ^13^C nuclear magnetic resonance (NMR) spectroscopy can been used to examine the compositions and polysaccharide mobilities in cell walls. The CP/MAS based relaxation time constants T_1ρ_^H^ (rotating-frame relaxation time constant of protons) and T_1_^C^ (spin-lattice relaxation time constant of ^13^C) are considered to be the most useful time constants for cell-wall samples not enriched in ^13^C [9]. By measuring their T_1ρ_^H^ and T_1_^C^ values the mobility of the individual polysaccharides can be determined and hence their contribution to the physical properties of the walls. Solid-state ^13^C NMR spectroscopy has been used to examine celery collenchyma cell walls [4, 8, 10–12]. This technique showed, for example that the (1 → 5)-α-l-arabinan side chains of RG1 in these cell walls are highly mobile. However, there have been no studies to investigate compositional changes in collenchyma cell walls during cell development.

There have also been few recent histochemical investigations to determine the locations of specific polysaccharides within collenchyma cell walls and no studies of collenchyma cell walls at different stages of development. Some early studies reported that collenchyma walls had a lamellar structure with lamellae rich in cellulose alternating with lamellae rich in non-cellulosic polysaccharides, in particular pectic polysaccharides. For example, bright-field microscopy with methylene blue staining of pectic polysaccharides showed a lamellar distribution in collenchyma cell walls of cow parsnip [5]. Bright-field light microscopy and transmission electron microscopy were also used to examine the distribution of pectic polysaccharides in the walls of different types of collenchyma cells in ten different species [13]. Bright-field light microscopy was used with the pectic polysaccharide stain ruthenium red and the alkaline hydroxylamine-ferric chloride staining reaction for pectic polysaccharides (the Reeve method) [14]. Transmission electron microscopy was used with an adaptation of the Reeve method [15]. With the bright-field light microscopy, a lamellar distribution of pectic polysaccharides was detected in only three species, including celery, but with transmission electron microscopy, equivocal results were obtained. Since these early studies, a range of monoclonal antibodies have been developed that will specifically recognize a number of common plant cell-wall polysaccharides, and immunofluorescence and immunogold microscopy have been used to determine the locations of these within cell walls [16, 17]. We are unaware of detailed studies with monoclonal antibodies that specifically target collenchyma cell walls of a particular species. However, because collenchyma cells occur commonly, some immunofluorescence information is available for several such monoclonal antibodies on a number of species and has been reviewed by Leroux [3]. This includes information on the locations of HG [3, 18, 19], the side chains of RG-I [3, 18, 19], xyloglucans [19], and heteroxylans [20], in stems of elderberry (*Sambucus* sp.) and tobacco (*Nicotiana tabacum*), and petioles of tomato (*Solanum lycopersicum*) fruit of unknown maturity.

Collenchyma cells in celery petioles elongate greatly during development and the stage of cell development is closely correlated with petiole length [21, 22]. Because the sub-epidermal collenchyma strands in celery petioles can easily be extracted, without contamination from other cell types, from petioles of different lengths, this is an excellent system to examine changes in only collenchyma cell walls during development, with the ultimate aim of relating these changes to the elongation process in this cell type.

In the present study, we first tested the hypothesis that all of the thick collenchyma walls are laid down while the cells are still elongating, i. e. the thick walls are indeed primary walls. Second, we investigated changes in cell-wall polysaccharide compositions at four different stages of collenchyma cell development by extracting collenchyma strands at these different stages, isolating cell walls and determining their monosaccharide compositions. We also used these isolated cell walls to obtain information about developmental changes in the mobilities of HG and cellulose by using solid-state ^13^C NMR spectroscopy. Third, we carried out immunofluorescence and immunogold microscopy with eight monoclonal antibodies that recognize glycan epitopes to determine the spatio-temporal locations of specific cell-wall polysaccharides at different developmental stages.

## Results

### Collenchyma cells elongate extensively during development and their walls thicken during elongation

**Fig. 1:**
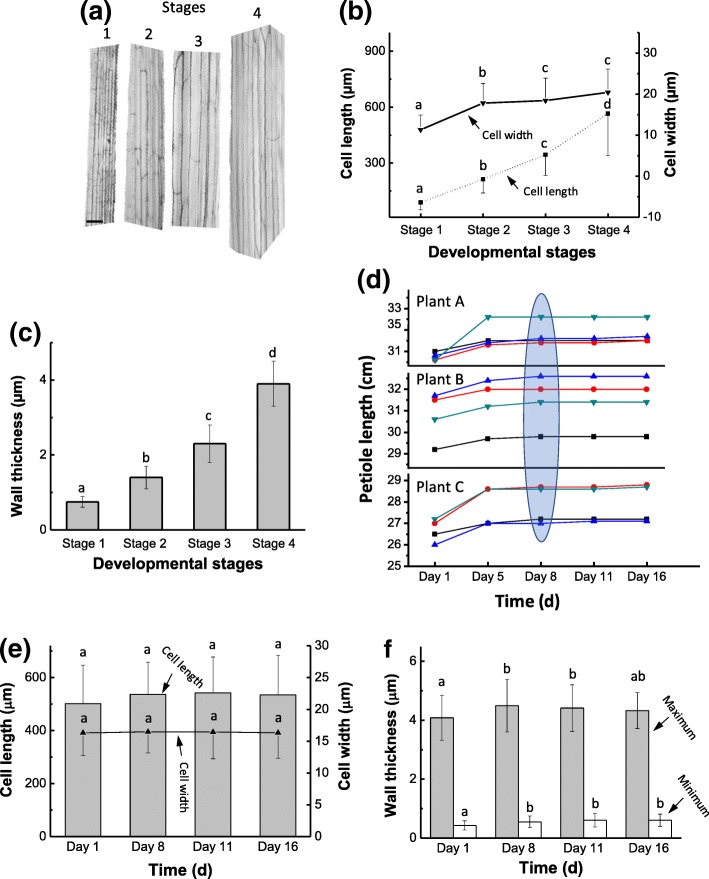
Structures of celery collenchyma cells at different developmental stages from petioles of different length. **a** Isolated collenchyma strands at developmental Stage 1 (2.8 cm long petiole), Stage 2 (12 cm petiole), Stage 3 (23 cm petiole), Stage 4 (38 cm petiole). Stained with 0.1% (w/v) aqueous toluidine blue, and color was adjusted to grey from original blue to better show the cell boundary. Scale bar= 50 μm. **b** Average collenchyma cell width () and length () at different developmental stages (n ≥180 cells per Stage). Stage 1-4, from 2-5 cm, 10-15 cm, 20-25 cm and 35-40 cm long petioles, respectively. **c** The average maximum wall thickness of collenchyma cells (n ≥ 30 per stage) at different developmental stages. Measurements were made on dehydrated cell walls. Stage 1-4, from 3 cm, 13 cm, 23 cm, 39 cm long petioles. **d** The elongation of petioles from three different plants (A, B and C) of “Triple 8” celery at a late stage of development. The ellipse highlighted the time when the petioles stopped elongating. Different symbols indicate different petioles. **e** The width () and length () of collenchyma cells from petioles before and after petioles stopped elongating. (n ≥ 45). **f** The average maximum () and minimum () wall thickness of collenchyma cells (n ≥ 45) from celery petioles before and after petioles stopped elongating. Different letters a, b, c, d indicate significant difference (*P <*0.05). The error bars indicate the standard deviation

**Fig. 2:**
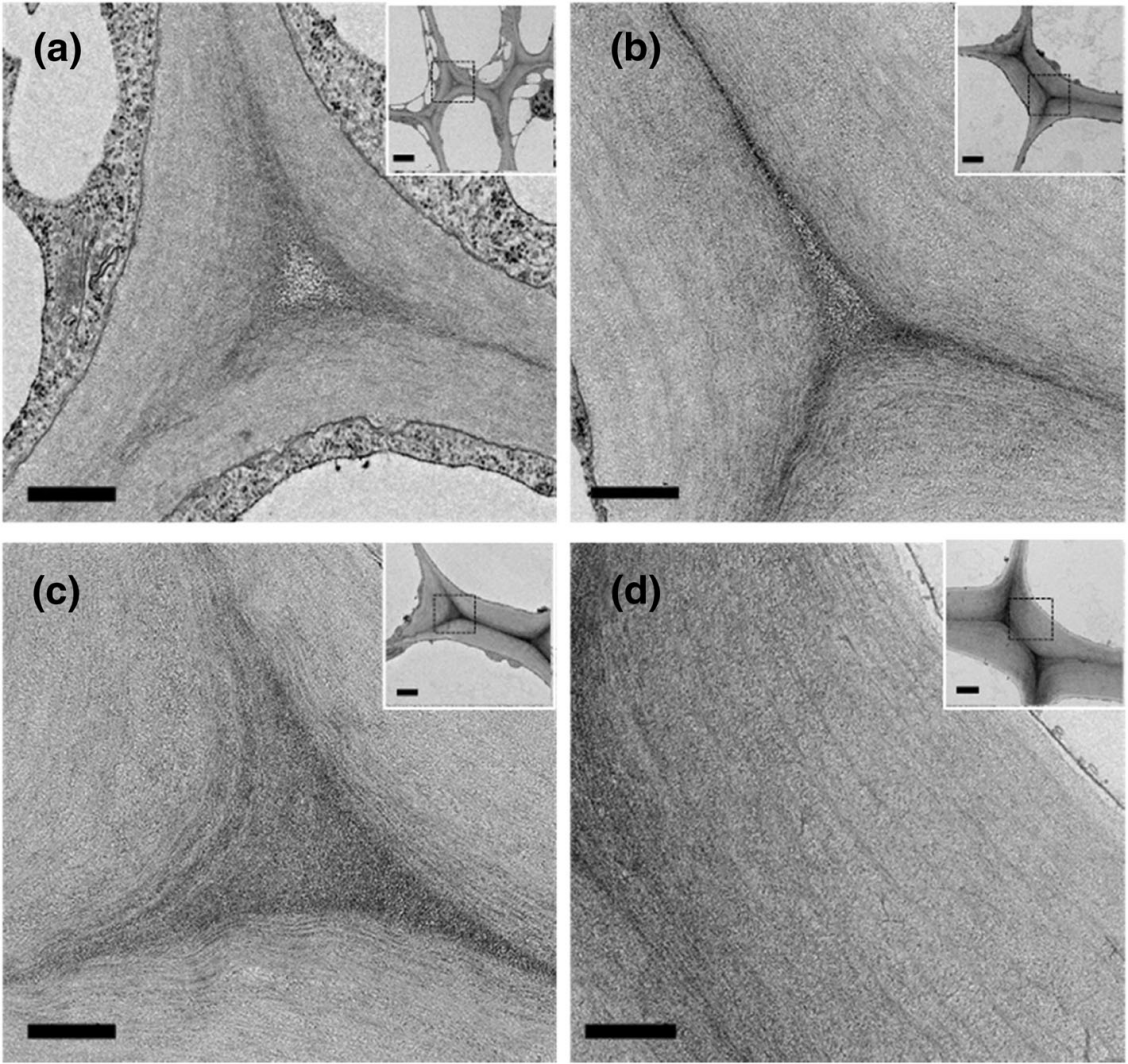
Transmission electron micrographs of transverse sections of celery collenchyma cells at four stages of development. **a**, Stage 1; **b**, Stage 2; **c**, Stage 3; **d**, Stage 4. The insets are low magnification images of the collenchyma cells and the rectangles show thickened walls at the cell corners used for the high magnification images in the main panels. These high magnification images show the polylamellate structure of the collenchyma cell walls. Scale bars= 500 nm (main panel) and 2 μm (insets)

Bright-field light microscopy showed that the celery collenchyma cells became wider and longer during development (Fig. 1a), with the width increasing markedly from Stage 1 to 2, but only slightly from Stage 2 to Stage 4 (Fig. 1b). However, their length continued to increase throughout development, with the increase greatest between Stages 3 and 4 (Fig. 1b). TEM images of transverse sections (Fig. 2) showed that at Stage 1 the collenchyma cell walls were thin with slight thickening at the cell corners (Fig. 2a), but at Stage 4 they had become very thick at the cell corners (Fig. 2d). An intercellular space is visible at Stage 1, and a small one is also present at Stage 2, but not at developmental Stages 3 and 4 (Fig. 2). The thickened walls are polylamellate because of cellulose microfibrils oriented at different angles (Fig. 2). The average maximum thickness of the dehydrated walls (measured at the cell corners) increased from 0.8 μm at Stage 1 to ~ 2.5 μm at Stage 3, to ~ 4.0 μm at Stage 4 (Fig. 1c).

To determine if the collenchyma cell walls continue to thicken after cell elongation ceases, celery petioles that had almost stopped growing were examined in detail. Petioles from three different plants (A, B and C) of the cultivar “Triple Eight” were measured and found to have elongated 0.5–1.5 cm from day 1 to 5, but had stopped elongating after day 8 (Fig. 1d). The lengths and widths of collenchyma cells from the same petioles were also measured, with each petiole sampled for cells only once (Fig. 1e). Cell length increased slightly from day 1 to 8, but no further increase occurred after this, indicating that elongation had ceased. Both maximum and minimum wall thickness increased from day 1 to day 8, but remained constant from day 8 to 16 (Fig. 1f), indicating that no wall thickening occurred after the cells had stopped elongating.

### The monosaccharide compositions of collenchyma cell walls change during development

The collenchyma cell walls had a high content of total uronic acids, regardless of stage of development (Table 1). The degree of methyl esterification of HG was similar at Stages 1 to 3, but dropped steeply by Stage 4. Cellulosic Glc accounted for approximately one third of the total monosaccharides, and the proportion increased from Stage 1 to 4, as did the proportions of Xyl and Man (Table 1). Approximately 9 mol% Ara and Gal occurred in the cell walls at Stage 1, but this had decreased considerably to 2.5 mol% and 4.7 mol%, respectively at Stage 4.

**Table 1:** The yield, monosaccharide compositions (mol%) and degree of methyl esterification of cell walls and HEPES fractions from celery collenchyma strands at different stages of development

Samples	Stage of development	Petiole length (cm)	Yield (%)	Monosaccharides									TS (μg/mg)	DM
				Rha	Fuc	Ara	Xyl	Man	Gal	Glc	C-Glc	UA		
CWs	1	2–5	7.1	2.2^d^	0.8^a^	9.1^d^	6.2^a^	2.0^a^	9.4^b^	2.0^a^	28.0^a^	40.3^b^	950^a^	36.1^b^
	2	10–15	7.8	1.9^c^	0.9^b^	6.5^c^	6.7^b^	2.6^b^	10.3^b^	2.1^b^	31.9^b^	37.0^a^	982^ab^	35.7^b^
	3	20–25	10.2	1.7^b^	0.9^b^	4.3^b^	7.7^c^	2.9^c^	9.5^b^	2.2^c^	33.7^c^	36.7^a^	951^a^	34.9^b^
	4	35–40	12.8	1.5^a^	0.9^b^	2.5^a^	7.9^c^	3.0^c^	4.7^a^	2.2^c^	35.8^d^	41.5^b^	1013^b^	19.2^a^
HEPES fraction	1	2–5	2.4	2.4^a^	4.6^c^	15.5^d^	9.9^a^	4.5^c^	22.8^c^	9.8^c^	ND	30.5^a^	183^a^	ND
	2	10–15	1.7	2.6^a^	1.1^b^	11.4^c^	12.9^b^	2.8^a^	16.5^b^	5.6^b^	ND	47.1^c^	436^b^	ND
	3	20–25	1.1	2.5^a^	0.9^ab^	10.1^b^	16.6^c^	3.0^b^	16.0^ab^	6.6^a^	ND	44.3^b^	420^b^	ND
	4	35–40	0.9	2.8^a^	0.6^a^	9.2^a^	17.3^d^	2.9^b^	14.9^a^	6.3^a^	ND	46.0^bc^	435^b^	ND

The yield of the CWs and HEPES fraction is the freeze-dried weight recovered from 100 g fresh weight of collenchyma strands

Rha and Glc values are from the 2 M TFA hydrolysis; all other neutral sugars values are from H_2_SO_4_ hydrolysis

Values are the average of duplicates or triplicates (*n* = 2 or 3)

Different letters (a, b, c) indicate significant differences (*P* ≤ 0.05)

*ND* not determined, *DM* degree of methyl esterification of pectin (mol%), *Rha* Rhamnose, *Fuc* fucose, *Ara* arabinose, *Xyl* xylose, man mannose, *Gal* galactose, *Glc* non-cellulosic glucose from TFA hydrolysis, *C-Glc* cellulose glucose, *TFA* glucose subtracted from H_2_SO_4_ glucose, *UA* uronic acids, *TS* total monosaccharides, sum of uronic acid and neutral monosaccharides

During the isolation of the collenchyma cell walls, a small proportion of the polysaccharides (≤ 2.4%) was soluble in the HEPES (4-(2-Hydroxyethyl)piperazine-1-ethanesulfonic acid) buffer, and this probably represented material in the apoplasts (Table 1). The monosaccharide composition of this HEPES soluble material indicated the presence of abundant uronic acids, followed by Ara, Gal and Xyl. The proportions of uronic acids increased from developmental Stages 1 to 4 as did the proportion of Xyl. However, the proportions of Ara, Gal and Fuc showed the opposite trend. This suggests the presence of a water-soluble heteroxylan [4].

### Immunofluorescence labelling of collenchyma cell walls at different developmental stages

**Fig. 3:**
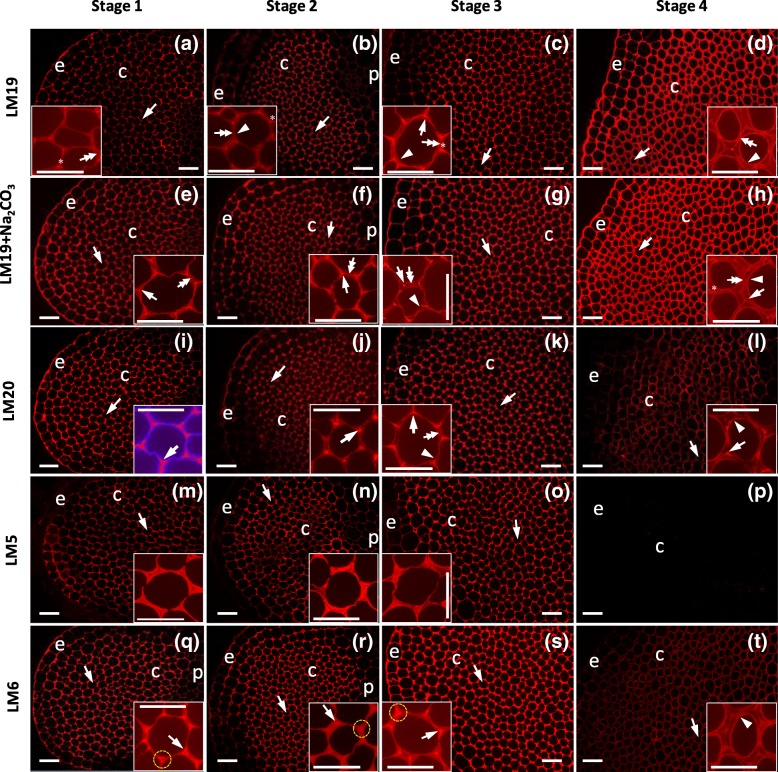
Immunofluorescence micrographs of transverse sections of collenchyma strands at four stages of development indirectly labelled with the monoclonal antibodies LM19 (**a**-**h**), LM20 (**i**-**l**), LM5 (**m**-**p**) and LM6 (**q**-**t**). LM19 + Na_2_CO_3_, sections were pretreated with 0.1 M Na_2_CO_3_ before labelling with LM19. Stage 1 (2.6 cm long petiole), (**a**, **e**, **i**, **m**, **q**); Stage 2 (11 cm long petiole), (**b**, **f**, **j**, **n**, **r**); Stage 3 (24 cm long petiole), (**c**, **g**, **k**, **o**, **s**); Stage 4 (40 cm long petiole), (**d**, **h**, **I**, **p**, **t**). The insets show the collenchyma cells marked with arrows in the main panel at higher magnifications. The intense labelling in the insets were marked with arrows, double headed arrows, arrowheads and double arrows. Arrows indicate the middle lamella junctions or cell corners, double headed arrows indicate the middle lamella, arrowheads indicate the inner region of the walls, and stars indicate the artefacts (strong labelling originates from collapse of walls). A dashed-line circle indicates the absence of labelling at the centre of a cell corner. e = epidermis, c = collenchyma cells, p = parenchyma cells. Scale = 50 μm in the main panel and = 25 μm in the insets

Monoclonal antibodies were used to localize the non- or low-methyl esterified HGs (with LM19), the more highly methyl esterified HGs (with LM20), and the RG-I side chains (1 → 4)-β-d-galactans (with LM5) and (1 → 5)-α-l-arabinans (with LM6). LM19 labelled collenchyma cell walls at all stages of development (Fig. 3a-d). At Stage 1, labelling was most abundant at the cell corners, and the middle lamellae were also particularly heavily labelled (Fig. 3 a). At Stage 2, a similar labelling pattern was found except slightly more labelling of the inner walls was observed (Fig. 3b). At Stage 3, LM19 labelling was again mainly at the cell corners, followed by the middle lamellae and inner walls (Fig. 3c). At Stage 4, labelling intensity was the highest, with only slightly more labelling in the middle lamellae and inner walls than in other structures (Fig. 3d). However, at Stages 1–3, some of the areas of strong labelling at the middle lamella junctions (regions in the cell corners where the middle lamellae of adjoining cells meet) (Fig. 3a-c) may be artefacts that most likely occurred after swelling of cell walls that were dehydrated during sample processing. These fluorescent areas have particularly sharp edges or irregular shapes. This effect was most obvious with the LM19 labelling. As expected, Na_2_CO_3_ pre-treatment caused an overall increase in LM19 labelling (Fig. 3e-h), indicating a significant reduction in the methyl esterification of HG. At Stages 1–2, LM20 showed extensive labelling at the cell corners, but it was difficult to see labelling of the middle lamellae at these stages (Fig. 3i-j). At Stage 3, in addition to the cell corners, labelling was abundant at the middle lamellae and present over the inner walls (Fig. 3k). At Stage 4, weaker labelling was observed, with most labelling on the inner walls, followed by the centres of cell corners (Fig. 3l). As expected, Na_2_CO_3_ pre-treatment resulted in a loss of LM20 labelling (Additional file 1: Figure S1). At Stages 1–3, the LM5 epitope was distributed throughout the collenchyma cell walls, but with very weak labelling of the middle lamellae and middle lamella junctions (Fig. 3m-o), whereas at Stage 4, no LM5 epitope was observed (Fig. 3p). At Stages 1–3, LM6 showed stronger labelling than LM5 throughout the walls and was more abundant in the outer walls adjacent to the middle lamella junctions (Fig. 3q-s). However, at Stage 4, LM6 labelling was markedly less and mostly located on the inner walls (Fig. 3t).

**Fig. 4:**
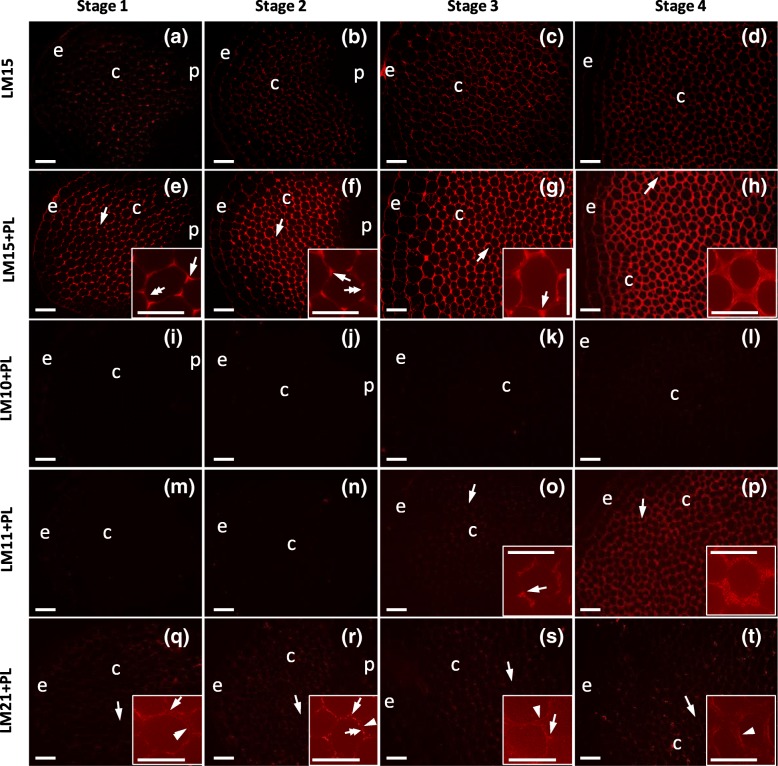
Immunofluorescence micrographs of transverse sections from celery collenchyma strands at four stages of development indirectly labelled with the monoclonal antibodies LM15, LM10, LM11 and LM21. LM15 (**a-d**), sections were labelled without pretreatment with pectate lyase before labelling with LM15; LM15+ PL (**e-h**), LM10+ PL (i-l), LM11+ PL (**m-p**), LM21+ PL (**q-t**), sections were pretreated with pectate lyase before labelling with LM15, LM10, LM11 and LM21, respectively. Stage 1 (2.6 cm long petiole) (**a**, **e**, **i**, **m**, **q**); Stage 2 (11 cm petiole) (**b**, **f**, **j**, **n**, **r**); Stage 3 (24 cm petiole) (**c**, **g**, **k**, **o**, **s**); Stage 4 (40 cm petiole) (**d**, **h**, **l**, **p**, **t**). The insets show the collenchyma cells marked with arrows in the main panel at higher magnifications. The intense labelling in the insets was marked with arrows, double headed arrows, arrowheads and double arrows. Arrows indicate the middle lamella junctions or cell corners, double headed arrows indicate the middle lamella, and arrowheads indicate the inner region of the walls, e = epidermis, c = collenchyma cells, p = parenchyma cells. Scale = 50 μm in the main panel. Scale = 25 μm in the insets

Monoclonal antibodies were also used to label xyloglucans (with LM15), heteroxylans (with LM10 and LM11) and heteromannans (with LM21). LM15 weakly labelled the collenchyma cell walls, with the labelling intensity increasing from Stage 1 to 4 (Fig. 4a-d). However, after pre-treatment with pectate lyase, the labelling intensity increased markedly (Fig. 4e-h), indicating that HG masked the LM15 epitopes. After pre-treatment, at Stages 1 and 2, LM15 labelling was mainly restricted to middle lamella junctions and the middle lamellae (Fig. 4e-f), whereas at Stage 3, labelling occurred throughout the cell corners (Fig. 4g) and at Stage 4 became noticeably more evenly distributed (Fig. 4h). With LM10, no labelling was observed at any stage, either before or after pectate lyase pre-treatment (Fig. 4i-l). At Stages 1 and 2, the LM11 epitope was not detectable in the walls (Fig. 4m-n), even after pectate lyase pre-treatment, but at Stage 3, weak labelling was found (Fig. 4o), and at Stage 4, stronger labelling was observed (Fig. 4p). Labelling was mainly located in the thickened regions of the walls, including cell corners. After pectate lyase pre-treatment, LM21 labelled collenchyma walls very weakly throughout development (Fig. 4q-t). At all stages, the antibody preferentially labelled the inner walls, but at Stages 1 and 2, it also labelled the cell corners (Fig. 4q-r). At Stage 2, the middle lamellae were also labelled. In control experiments, without pectate lyase pre-treatment, no labelling was observed with LM10, LM11 or LM21 (Additional file 1: Figure S1). In all the immunofluorescence labelling experiments, with all monoclonal antibodies and at all the stages, no labelling was observed in control experiments in which the primary antibody was omitted (Additional file 2: Figure S2 and Additional file 3: Figure S3).

### Immunogold labelling of collenchyma cell walls at different developmental stages

**Fig. 5:**
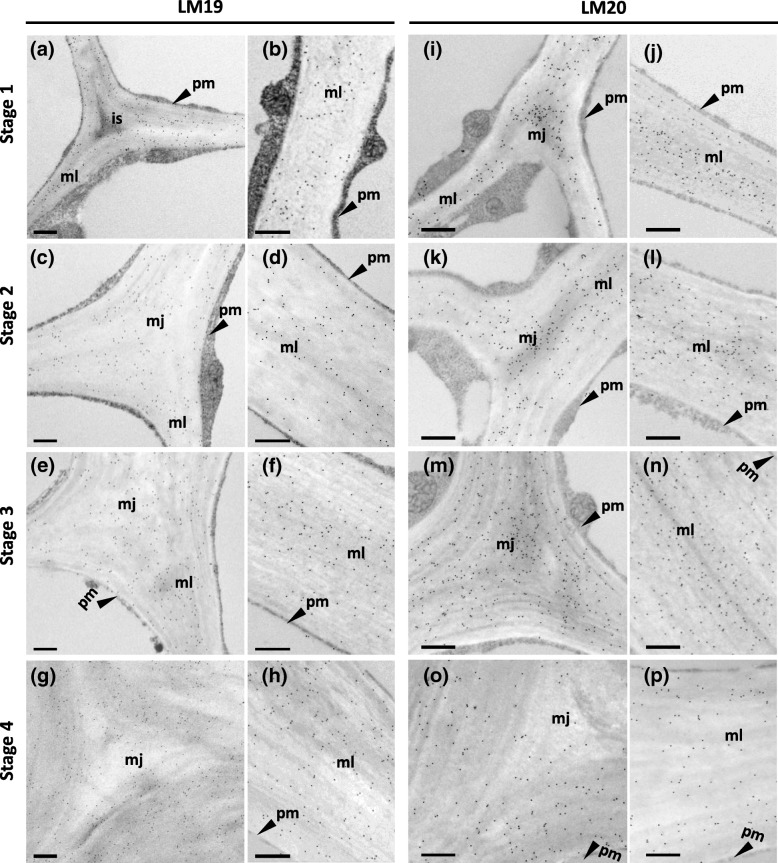
Immunogold micrographs of transverse sections of collenchyma strands at different developmental stages labelled with monoclonal antibodies LM19 (**a**-**h**) and LM20 (**i**-**p**). Stage 1 (2.6 cm long petiole) (**a**, **b**, **I**, **j**), Stage 2 (11 cm petiole) (**c**, **d**, **k**, **l**), Stage 3 (24 cm petiole) (**e**, **f**, **m**, **n**), Stage 4 (40 cm petiole) (**g**, **h**, **o**, **p**). Two different regions of cells were shown, cell corners (**a**, **c**, **e**, **g**, **i**, **k**, **m**, **o**) and thick walls (**b**, **d**, **f**, **h**, **j**, **l**, **n**, **p**) that connect two cells, is = intercellular space, mj = middle lamella junctions, ml = middle lamella, pm = plasma membrane. Scale = 500 nm

**Fig. 6:**
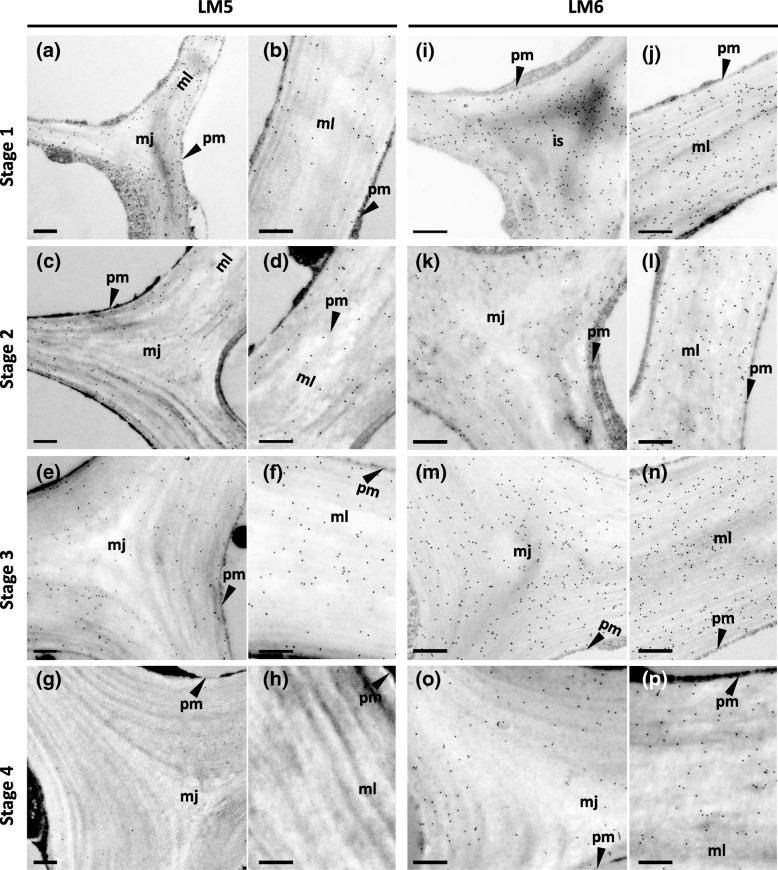
Immunogold micrographs of transverse sections of celery collenchyma strands at different developmental stages labelled with monoclonal antibodies LM5 (**a**-**h**) and LM6 ( **i**-**p**). Stage 1 (2.6 cm long petiole) (**a**, **b**, **I**, **j**), Stage 2 (11 cm petiole) (**c**, **d**, **k**, **l**), Stage 3 (24 cm petiole) (**e**, **f**, **m**, **n**), Stage 4 (40 cm petiole) (**g**, **h**, **o**, **p**). Two different regions of cells were shown, cell corners (**a**, **c**, **e**, **g**, **i**, **k**, **m**, **o**) and thick walls (**b**, **d**, **f**, **h**, **j**, **l**, **n**, **p**) that connect two cells, is = intercellular space, mj = middle lamella junctions, ml = middle lamella, pm = plasma membrane. Scale = 500 nm

**Fig. 7:**
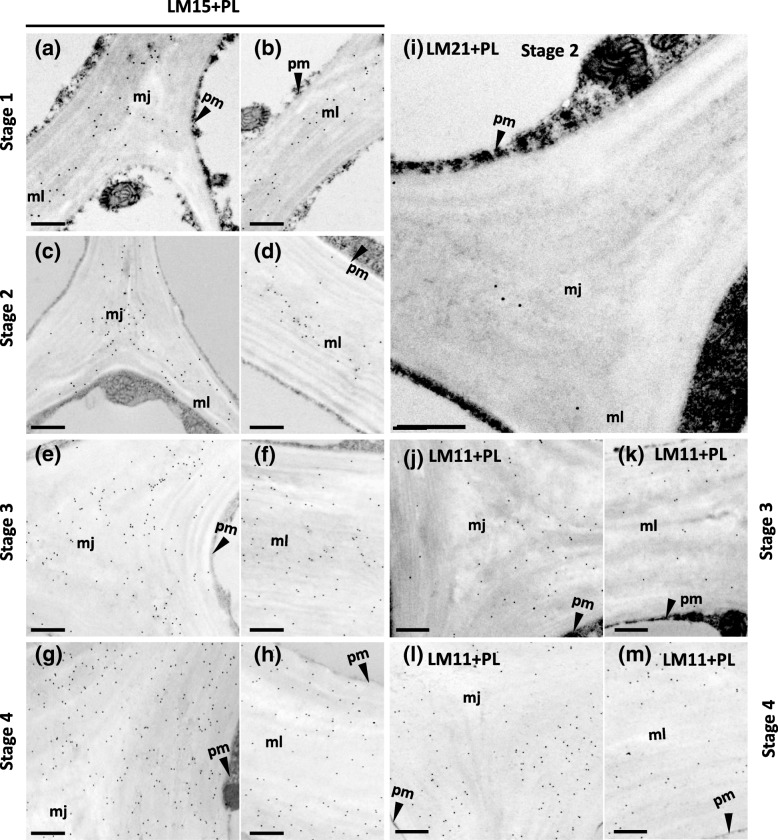
Immunogold micrographs of pectate lyase pretreated transverse sections of celery collenchyma strands at different developmental stages labelled with monoclonal antibodies LM15 (**a**-**h**), LM21 (**i**) and LM11 (**j**-**m**). Stage 1 (2.6 cm long petiole) (**a**, **b**), Stage 2 (11 cm petiole) (**c**, **d**, **i**), Stage 3 (24 cm petiole) (**e**, **f**, **j**, **k**), Stage 4 (40 cm petiole) (**g**, **h**, **l**, **m**). Two different regions of cells were shown, cell corners (**a**, **c**, **e**, **g**, **I**, **j**, **l**) and thick walls (**b**, **d**, **f**, **h**, **k**, **m**) that connect two cells, mj = middle lamella junctions, ml = middle lamella, pm = plasma membrane. Scale = 500 nm

Immunogold labelling of collenchyma cell walls showed similar patterns to those found with immunofluorescence labelling. Labelling of the cell corners and thickened walls are shown in Fig. 5, 6 and 7 and labelling of the thin walls in Additional file 4: Figure S4. At Stages 3 and 4, labelling of HG with LM19 was slightly greater than at Stages 1 and 2 (Fig. 5a-h**,** Additional file 4: Figure S4). At Stages 1–3, labelling occurred at the cell corners and all across the thickened walls, with more labelling in the middle lamellae and the adjacent outer walls (Fig. 5a-f**,** Additional file 4: Figure S4b-d). At Stage 4, labelling was more evenly distributed, but there appeared to be more gold particles over the middle lamellae and the adjacent outer walls (Fig. 5g-h, Additional file 4: Figure S4e). At Stages 1–3, LM20, which is specific for more highly methyl esterified HG, strongly labelled the middle lamella junctions, middle lamellae and the adjacent outer walls (Fig. 5i-n, Additional file 4: Figure S4f-h), but at Stage 4, the middle lamella junctions were only lightly labelled, and the rest of the walls were less strongly labelled (Fig. 5o-p**,** Additional file 4: Figure S4i). In particular, the thin walls showed only low intensity labelling, so that labelling locations are difficult to determine, but there was some labelling of the inner walls. At Stages 1–3, labelling with LM5, which is specific for (1 → 4)-β-d-galactans, showed little or no labelling at the middle lamellae and middle lamella junctions, but the rest of the thickened walls were evenly labelled (Fig. 6a-f**,** Additional file 4: Figure S4). However, in the thin regions of the walls, labelling was mainly restricted to the inner walls (Additional file 4: Figure S4j-l). At Stage 4, LM5 labelling was absent except for an occasional particle (Fig. 6g-h**,** Additional file 4: Figure S4 m). Labelling with LM6, which is specific for (1 → 5)-α-l-arabinans, was greater than with LM5, and, at Stages 1–3, this labelling was evenly distributed across the thickened walls, with slightly more labelling in the outer walls adjacent to the intercellular space (Stage 1) or middle lamella junctions (Stages 2–3) (Fig. 6i-p). At Stage 4, the labelling was much reduced, and distributed mostly over the inner walls (Fig. 6o-p, Additional file 4: Figure S4q). At Stages 1–3, in the thin regions of the walls, LM6 label was more intense at the middle lamellae (Additional file 4: Figure S4n-p), but at Stage 4 was mainly located on the inner walls (Additional file 4: Figure S4q).

After pectate lyase pre-treatment, the amount of labelling with LM15, which is specific for xyloglucan, increased with stage of development (Fig. 7a-h**,** Additional file 4: Figure S4r-u). At Stages 1 and 2, labelling was mainly at the middle lamellae and middle lamella junctions (Fig. 7a-d, Additional file 4: Figure S4r-s). However, at Stage 3, labelling was more widespread occurring throughout the cell corners and thickened walls (Fig. 7e-f, Additional file 4: Figure S4 t), and at Stage 4 was almost evenly distributed (Fig. 7g-h, Additional file 4: Figure S4u). As no immunofluorescence labelling with LM11, which is specific for heteroxylans, was observed at Stages 1 and 2, even after pectate lyase pre-treatment, no immunogold labelling was carried out at these stages. However, at Stage 3, gold labelling was mainly restricted to outer walls adjacent to middle lamella junctions (Fig. 7j, k). At Stage 4, there was more labelling which was widely distributed in the cell corners and thickened walls (Fig. 7l-m). Because immunofluorescence labelling with LM21, which is specific for heteromannans, was strongest at Stage 2, only this stage was further investigated using immunogold labelling. However, only a few gold particles were observed (Fig. 7i).

In control immunogold labelling experiments in which the primary antibodies were omitted, gold particles were very rarely found, both in untreated sections and in sections pre-treated with pectate lyase (Additional file 5: Figure S5 and Additional file 6: Figure S6, respectively).

### Mobilities of cellulose and homogalacturonan decrease during development

**Fig. 8:**
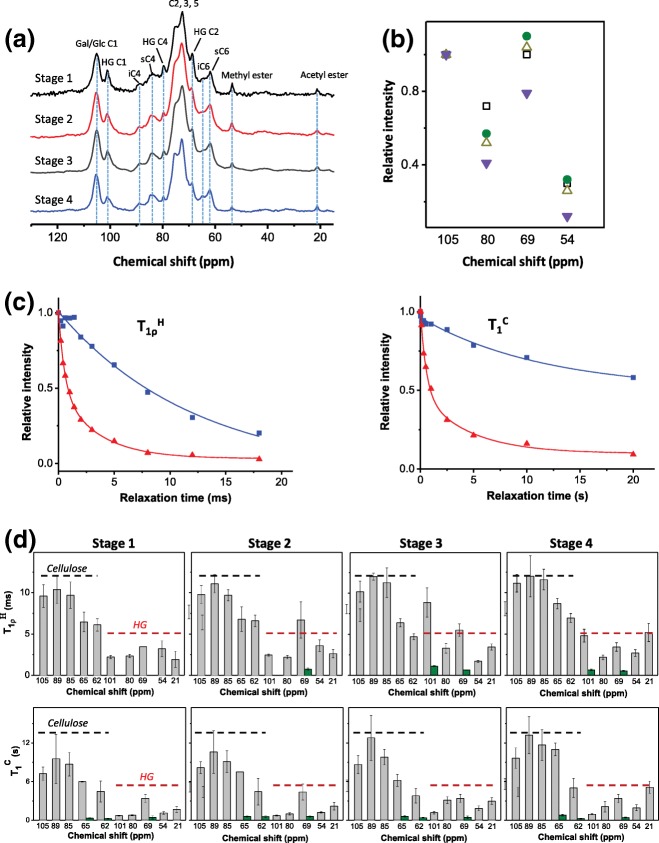
CP/MAS spectra and relaxation time constants of wall polysaccharides from celery collenchyma strands at four stages of development. Stage 1 (2-5 cm petioles), Stage 2 (10-15 cm petioles), Stage 3 (20-25 cm petioles), Stage 4( 35-40 cm petioles). **a** CP/MAS spectra of collenchyma cell walls with 65% (w/w) hydration. Gal/Glc C1, C-1 of galactose from galactans and glucose from cellulose; HG C1, C-1 of galacturonic acid from HG; iC4, C-4 of glucose from interior cellulose; sC4, C-4 of glucose from surface cellulose; HG C4, C-4 of galacturonic acid from HG. C2, 3, 5, carbon 2, 3, 5 of hexoses and galacturonic acid; HG C2, C-2 of galacturonic acid from HG; iC6, C-6 of cellulose from interior cellulose; sC6, C-6 of cellulose from surface cellulose. **b** Relative intensities of HG dominant signals from CP/MAS spectra, resulting from the absolute intensity of HG related signals divided by those of cellulosic signals at 105 ppm. Stage 1 (), Stage 2 (), Stage 3 (), Stage 4 (). **c** Representative relaxation pattern of T_1ρ_^H^ and T_1_^C^ from mainly cellulose at 89 ppm ( ) and HG at 69 ppm () in the collenchyma cell walls at Stage 4. **d** T_1ρ_^H^ and T_1_^C^ values of cellulose and HG in the collenchyma cell walls at different developmental stages. The chemical shifts beneath dashed line were clustered by cellulose (black line) and HG (red line). The dashed line set as guidance for comparison. Long and short components of the bi-exponentially decayed signals were shown as gray () and green () columns, respectively. The error bars were standard deviations from curve fitting using Origin software (see Methods)

The CP/MAS NMR spectra of collenchyma cell-wall preparations at different developmental stages are typical of primary walls of eudicotyledons and are dominated by signals from cellulose and semi-rigid pectic polysaccharides [23–25]. During development, the signals at 89 (C-4 Glc) and 65 ppm (C-6 Glc) from interior cellulose molecules become clearer (Fig. 8a). Pectic polysaccharide signals in CP/MAS are mainly from HG, with little contribution from the RG-I and RG-II backbones due to their small proportions in collenchyma cell walls. The (1 → 5)-α-l-arabinan and (1 → 4)-β-d-galactan side chains of RG-I are not detected in CP/MAS experiments due to their high flexibility. The intensities of the dominant peaks from HG at 80 ppm (C-4 GalA), 69 ppm (C-2 GalA) and 54 ppm (methyl esters) show some reduction during development (Fig. 8a). To quantify the change, the intensity of the cellulose peak at 105 ppm (C-1 Glc) was set as the internal standard, and the relative intensities of the peaks at 80 ppm were found to decrease consistently from Stages 1 to 4 (Fig. 8b). At 69 and 54 ppm, the relative intensities were similar at Stages 1–3, but were significantly higher than those at Stage 4 (Fig. 8b). This may be due to different proportions of cellulose and HG in the cell walls, and/or to differences in their mobilities. To investigate if their mobilities changed, measurements of the relaxation time constants T_1ρ_^H^ and T_1_^C^, representing movement in microseconds and nanoseconds, respectively, were performed on the collenchyma cell walls at the different developmental stages. The dipolar interaction can affect the T_1ρ_^H^ relaxation behaviour but this is only applicable to rigid crystal samples, which is not the case here. The relaxed spectra are shown in Additional file 7: Figure S7. On plotting the correlation between relative signal intensity versus the relaxation time, cellulose was found to relax slower than HG in the cell walls at all stages of development, and a representative example is shown in Fig. 8c.

Cellulose showed long T_1ρ_^H^ and T_1_^C^ values (Fig. 8d), and these increased gradually from Stages 1 to Stage 4, especially for C-1 and C-4, suggesting that cellulose becomes more rigid during development. At Stage 3, a slight reduction in T_1ρ_^H^ and T_1_^C^ was found for C-6 of cellulose. Values for T_1ρ_^H^ and T_1_^C^ of HG were variable, but nearly all were shorter than those of cellulose, although the longer components in the bi-exponential decay of 69 and 101 ppm in T_1ρ_^H^ showed some exceptions. Values for T_1ρ_^H^ of acetyl esters (21 ppm) increased gradually during development, whereas values for T_1ρ_^H^ of other HG resonances such as 80 ppm and 54 ppm remained relatively stable. Unlike T_1ρ_^H^, the T_1_^C^ of methyl (54 ppm) and acetyl (21 ppm) esters increased during development. In addition, at Stages 3 and 4, 101 and 80 ppm also showed longer T_1_^C^ than at Stages 1 and 2. However, not all of the T_1ρ_^H^ and T_1_^C^ of ^13^C resonances exactly follow the trend of having a longer value (being more rigid) at greater maturity.

## Discussion

### Mature collenchyma cells in celery petioles have thickened primary walls

Our results indicate that the walls of mature sub-epidermal collenchyma cells in celery petioles can be described as thickened primary walls. No evidence was obtained for the cell walls increasing in thickness after the cells had stopped elongating. However, the results obtained in celery may not necessarily apply to collenchyma cells in other species. Nevertheless, our results from celery are consistent with the findings of Majumdar and Preston [5] for the collenchyma cell walls of cow parsnip.

### Temporal variation in collenchyma cell-wall epitopes/ polysaccharide distributions

Our analytical and immunomicroscopy results indicate that the abundance and fine-structures of polysaccharides, particularly the pectic polysaccharides, change during development. A decrease in the degree of methyl esterification of HGs was shown analytically, and is consistent with the increased labelling by LM19 and the decreased labelling by LM20. Similar changes in pectic polysaccharides during cell elongation have been reported using analytical methods and immunomicroscopy in organs of the eudicotyledon *Arabidopsis thaliana* containing mostly parenchyma and epidermal cells with primary walls. For example, an analytical study on methyl esterification of HG in hypocotyl cell walls showed that higher degrees of methyl esterification were associated with higher rates of cell elongation [26]. A detailed immunofluorescence microscopy study of *A. thaliana* walls of different cell types at three developmental stages in stems showed a decline in labelling with LM20 and increase in labelling with LM19 occurred during maturation, particularly in the epidermal cell walls [27]. Such changes in the methyl esterification of HG may affect cross linking of adjacent HG molecules. Adjacent HG molecules that have at least ten consecutive galacturonosyl residues that are not methyl esterified can form calcium ionic cross bridges, the so-called “egg-box” structures [28], resulting in calcium pectate junction zones and stiffer cell walls [29]. Methyl esterification of HGs prevents the formation of these cross bridges, allowing relative movement of adjacent HG molecules, resulting in more flexible molecules favouring cell-wall expansion [26, 30]. HGs are synthesized in Golgi bodies and then deposited into cell walls via Golgi vesicles. They initially have a high degree of methyl esterification [31], but the methyl groups are progressively removed in the cell walls by the actions of pectin methylesterases (PME). This can lead either to the formation of calcium pectate junction zones, as indicated above, or the degradation of the non-methyl esterified HG by polygalacturonases [30]. In the present study, we found no significant changes during development in the total uronic acid content of the cell walls, indicating there was no significant degradation of non-methyl esterified HG. However, the decrease in mobility of HG as determined using solid-state NMR spectroscopy is consistent with the formation of calcium pectate junction zones.

Our immunofluorescence and immunogold microscopy results indicate that the (1 → 5)-α-l-arabinan and (1 → 4)-β-d-galactan side chains of RG-I also decreased in proportion during development of the collenchyma cell walls. These results also indicate that (1 → 5)-α-l-arabinan side chains predominated over (1 → 4)-β-d-galactan side chains throughout development. Linkage analysis of the polysaccharides in collenchyma cell walls from fully elongated petioles (Stage 4) showed this predominance of (1 → 5)-α-l-arabinan side chains [4]. The decrease in the proportion of RG-I side chains could result either from fewer side chains being present on newly synthesized RG-1 or from the partial enzymatic degradation of these side chains. Previous research on other eudicotyledon primary walls showed that in dividing cells there were higher proportions of (1 → 5)-α-l-arabinan side chains relative to (1 → 4)-β-galactan side chains, whereas in elongating cells the opposite was found. For example, immunofluorescence microscopy studies showed that (1 → 5)-α-l-arabinan, recognized by LM6, was located in the walls of dividing cells in carrot (*Daucus carota*) root apices [32] and *A. thaliana* roots [33], whereas (1 → 4)-β-d-galactan, recognized by LM5, was located in primary walls of elongating cells in potato (*Solanum tuberosum*) stolons [34]. When cell expansion stopped or slowed down in these plants, LM6 and/or LM5 labelling declined [32–34]. Little is known about the specific roles of (1 → 5)-α-l-arabinan and (1 → 4)-β-d-galactan side chains in primary cell walls, but modulation of their proportions is likely to affect the wall mechanics. Both types of side chains are known to be highly mobile and are likely to confer the water-binding properties on pectic polysaccharides [35]. In addition, a small proportion of these side chains are non-covalently linked to cellulose microfibrils [36]. Previous studies suggested that (1 → 5)-α-l-arabinans contribute to the flexibility of parenchyma cell walls [37–40]. Thus, the presence of higher proportions of (1 → 5)-α-l-arabinan RG-I side chains at early developmental stages of celery collenchyma walls would help to maintain wall flexibility favouring wall expansion.

From the analytical data, the proportion of cellulose in celery collenchyma cell walls increased during development. Immunofluorescence and immunogold microscopy with LM15 and LM11, which specifically detect xyloglucans and heteroxylans, respectively, also indicated some increase in the proportions of these polysaccharides during development. Such increases could contribute to more coherent, stiffer walls, probably by these polysaccharides cross-linking cellulose microfibrils. The lower proportions of cellulose, xyloglucans and heteroxylans at earlier stages of development may result in more flexible walls favouring cell elongation. Although the proportion of mannose increased significantly in the monosaccharide compositions of cell walls from Stage 1 to Stage 4, immunofluorescence microscopy showed the most labelling at Stage 2, but the labelling was weak at all developmental stages and it is difficult to determine if changes in heteromannan occurred.

### Spatio-temporal distributions of collenchyma cell-wall epitopes/ polysaccharides

Immunogold microscopy with LM19 and LM20 showed no evidence of the lamellar distribution of HG pectic polysaccharides in celery collenchyma walls in contrast to a histochemical study using ruthenium red and the alkaline hydroxylamine-ferric chloride staining [13]. However, the immunogold micrographs showed alternating electron lucent and dense lamellae in the collenchyma walls (e.g. Fig. 5), which may result from cellulose microfibrils oriented at different angles (see also Fig. 2). Another monoclonal antibody, JIM5, which like LM19 also recognizes HGs that are non- or low-methyl esterified, also labelled collenchyma cell walls in tomato petioles, but no lamellar distribution was found using immunofluorescence microscopy [18]. Overall, although LM19 and LM20 labelling occurred throughout the celery collenchyma walls, labelling of the middle lamellae was predominant, both in the immunofluorescence and immunogold labelling, which is consistent with high proportions of HG being present at this location throughout development, but with a reduction in the degree of methyl esterification occurring during development. The results suggest that early in development, the methyl esterification will limit the formation of HG-Ca^2+^ bridges (see above) at the middle lamellae and middle lamella junctions, allowing adjacent young cells to slide relative to one another, promoting cell elongation [41]. At maturity (Stage 4), the reduction in LM20 labelling and increase in LM19 labelling at these locations indicates much less methyl esterification resulting in an enhancement of HG-Ca^2+^ bridges, preventing movement between adjacent cells.

Unlike HG, using immunofluorescence and immunogold microscopy with LM5 the (1 → 4)-β-d-galactan side chains of RG-1 were localized in the walls of the celery collenchyma cells rather than the middle lamellae and middle lamella junctions. In contrast, using LM6 the (1 → 5)-α-l-arabinan side chains were found in the middle lamellae and middle lamella junctions as well as the rest of the cell walls. The distributions of both types of side chain did not change during cell elongation (Stages 2–3), but at maturity when elongation had ceased (Stage 4), there were no (1 → 4)-β-d-galactan side chains detected by LM5 labelling. However, some (1 → 5)-α-l-arabinan side chains were present, but mainly in the inner wall region as indicated by the LM6 labelling. Immunofluorescence microscopy of collenchyma cell walls with LM5 has previously been reported for tomato petiole [18] and elderberry (*Sambucus nigra*) stem [3]. In both species, the labelling was confined to the inner wall region; but the stage of development of the collenchyma cells was not reported.

Immunofluorescence and immunogold microscopy with LM15, which is specific for xyloglucans, showed that at the earlier stages of development (Stages 1–3), it was mainly restricted to the middle lamellae and middle lamella junctions; this was particularly well shown by the immunogold labelling. Immunofluorescence microscopy with LM15 of the walls of collenchyma cells, at an unknown stage of development, has previously been reported for tomato (*Solanum lycopersicum*) stems, where the labelling was found mainly in the walls adjacent to the middle lamellae [19]. An earlier study with LM15 using immunofluorescence microscopy [42] and with CCRC-M86, another monoclonal antibody that recognizes xyloglucans, using immunofluorescence and immunogold microscopy [43], also showed xyloglucans were located at the middle lamellae in the parenchyma cells of the pericarp of unripe tomato fruit [42, 43]. These observations suggest xyloglucans may play a role in cell-cell adhesion in both collenchyma and parenchyma cells [19, 44], possibly by covalently binding to pectic polysaccharides [45]. Later in the development of celery collenchyma cells (Stage 4), the xyloglucans become more widely distributed in the wall and may be involved in maintaining the structures of these thicker walls.

Immunofluorescence microscopy with LM11, which is specific for heteroxylans, detected labelling only late in development, where immunogold microscopy showed it occurred over the thickened regions of the celery collenchyma walls rather than over the middle lamellae. This suggests heteroxylans have different functional roles in the collenchyma walls compared with xyloglucans. Heteroxylans have previously been detected with LM10 and LM11 using immunofluorescence microscopy in collenchyma cell walls of tobacco (*Nicotiana tabacum*) [20], where they were located in the inner and outer regions, respectively, of the walls at cell corners. In celery collenchyma walls, labelling was seen with LM11, but not with LM10, suggesting the heteroxylans are quite highly substituted. However, using immunofluorescence microscopy, a long exposure time was needed to detect this labelling. In our earlier study [4], glycosyl linkage analysis of walls isolated from fully elongated celery collenchyma cells showed that the heteroxylans were ~ 20% substituted. The wider distribution of heteroxylans in the collenchyma walls at Stage 4 compared with Stage 3, found using immunogold microscopy, suggests they, like xyloglucans, may also contribute to maintaining the intactness of the walls.

### Cellulose and homogalacturonan in collenchyma walls become less mobile during development

The relaxation time constants T_1_^C^ and T_1ρ_^H^ for cellulose in collenchyma walls showed that it becomes more rigid during development. Cellulose is the most rigid component in primary cell walls, and its rigidity can be affected by self-aggregation and cross-linking with other cell-wall components [12, 23, 46]. Increases in the proportions of xyloglucans and heteroxylans during development may be accompanied by more of these polysaccharides binding to cellulose and that may account for some of the increased rigidity of cellulose. Increased relaxation time constants T_1ρ_^H^ and T_1_^C^ of the dominant HG signals indicated that HGs also become more rigid during development, although to a lesser extent than cellulose. This may be the result of the formation of ionic bonding through calcium ions, creating more rigid pectate gels [29, 47]. The increases during development in T_1_^C^ values of the methyl ester and acetyl groups of HG are probably also the result of the formation of more rigid pectate gels. During collenchyma development, T_1ρ_^H^ decay of HG resonances at 101 and 69 ppm changed from mono-exponential to bi-exponential, suggesting increased heterogeneity of the HG microenvironment [48] in the cell walls. In contrast, the presence in T_1_^C^ of fast and slow components at C-6 (65, 62 ppm) of cellulose probably reflects mobility heterogeneity of cellulose molecules located at both the surface and interior of cellulose microfibrils, originating from their different conformations [49] or the interaction with non-cellulosic wall polysaccharides [50]. We did not investigate the variation in the mobilities of RG-I side chain (1 → 5)-α-l-arabinan and (1 → 4)-β-d-galactan during collenchyma development because they are very flexible and too mobile to be measured reliably by CP/MAS. Direct polarization has been found to be effective in gathering signals from these mobile side chains [4], but it is impractical to use for the relaxation time measurements due to their overlapping with signals from rigid and semi-rigid structures.

## Conclusions

Our research confirmed celery collenchyma walls are exclusively primary cell walls. A combination of analytical studies and immunomicroscopy on celery collenchyma cell walls at four stages of development showed there were changes during development in the proportions, fine structures and locations of the component cell-wall polysaccharides. Using solid-state NMR spectroscopy, we also showed that particularly cellulose, but also HGs, decreased in mobilities during development, which is consistent with the collenchyma cell walls becoming stiffer as elongation stopped.

## Methods

### Plant materials

Celery (*Apium graveolens* L*.*, ‘Tango’) was grown from seed in an unheated glasshouse at Clark Nurseries Ltd., Pukekohe, New Zealand (geographical location 37° 20’ S, 174° 88′ E), and harvested after six months of growth. Petioles were detached, washed and categorized into four stages of development based on petiole length (recorded from the base to the first leaflet junction): Stage 1 (2–5 cm), Stage 2 (10–15 cm), Stage 3 (20–25 cm) and Stage 4 (35–40 cm). The collenchyma cells at the different stages had different lengths and wall thicknesses. Previous studies have shown that petiole length in celery is closely correlated with collenchyma cell development [22]. Petioles at Stages 1 and 2 were obtained from the inner region of the bundle, petioles at Stage 3 were from the middle of the bundle, and petioles at Stage 4 were from the outer region of the bundle. Sub-epidermal collenchyma strands were taken from either an entire petiole (for compositional analysis) or from midway along the petiole (for all other work).

To determine if collenchyma wall thickening continued to occur after the cells had stopped elongating, petioles were examined from another cultivar, ‘Triple Eight’, grown for four months at the same location as cultivar ‘Tango’, but outdoors. It was assumed the collenchyma cells in the two cultivars behaved similarly. Petioles examined were almost fully elongated, but were still elongating. Three celery plants were used (A, B, C). On each plant, four petioles (petioles 1, 2, 3, 4, lengths 26–33 cm) were selected from the outer region of the bundle. On day 1, marks were made at intervals of 5 cm along the petioles with a waterproof, fine-tipped black marker pen. On the same day, a collenchyma strand was taken from the midway portion of petiole 1 from each plant and was processed (see below) to measure collenchyma cell length, width and wall thickness. The distances between the marks were recorded on days 5, 8, 11 and 16, and collenchyma strands were taken as before from petioles 2, 3, and 4 on days 8, 11 and 16, respectively. The lengths of petioles used to sample collenchyma strands continued to be measured after sampling.

### Measurement of the lengths and widths of collenchyma cells

Collenchyma strands (~ 5 mm long) from midway along petioles at the four different developmental stages were carefully detached using forceps, stained with an aqueous toluidine blue O solution (0.1%, w/v) and viewed by bright-field light microscopy as described by Chen et al. [4]. Cell lengths and widths at the different developmental stages were measured from the images using Image J (NIH, New York, USA). In each collenchyma strand for both cultivars, 15–20 cells were examined. Mean lengths and widths (± SD) were determined. Statistical analysis was conducted in SPSS Statistics 22 (IBM Corporation, Armonk, NY) using one-way ANOVA. *P* ≤ 0.05 was considered as statistically significant.

### Measurement of collenchyma cell-wall thickness

Collenchyma strands (~ 5 mm long) from midway along petioles were cut transversely into short segments (1–2 mm long), fixed with 100 mM potassium phosphate buffer (pH 7.4) containing 2.5% (w/v) glutaraldehyde for 2 h, washed with the same buffer, then post fixed with 1% (w/v) aqueous OsO_4_ for 2 h. After washing with buffer, the segments were dehydrated in an aqueous ethanol series (30, 50, 70, 85, 90, 100% twice), infiltrated with acetone for 5 min, a mixture of acetone and the epoxy resin Procure 812 (ProSciTech, Thuringowa Central, QLD, Australia) (1:1, v/v) for 1 h and pure resin for 24 h on a rotor. The segments were placed into fresh resin and polymerized at 60 °C for 48 h. Transverse sections (70 nm thick) were cut with an ultra-microtome (Model EM UC6, Leica, Vienna, Austria) using a diamond knife, collected on 200 mesh copper grids (ProSciTec), stained with 2% (w/v) aqueous uranyl acetate for 20 min and lead citrate [51] for 4 min before being examined with a transmission electron microscope (Model Technai 12; FEI, Hillsboro, OR, USA) at an accelerating voltage of 120 kv. The resulting images were used to measure collenchyma cell-wall thickness. At each developmental stage of cultivar ‘Tango’, the maximum wall thickness of 10–12 collenchyma cells was measured. For cultivar ‘Triple Eight’, both maximum and minimum wall thickness of ~ 15 cells was measured in each collenchyma strand. Statistical analysis of the data was conducted as above.

### Isolation of cell walls and determination of monosaccharide compositions

Cell walls were isolated as described in Melton and Smith [52] with modifications [4]. Briefly, collenchyma strands from petioles of different lengths (Stages 1–4; see above) were homogenized in buffer, cell walls were collected on nylon mesh, washed, dialyzed and freeze-dried. Cell walls were hydrolyzed with 2 M trifluoroacetic acid (TFA) [53, 54] or by a two-stage procedure using H_2_SO_4_ [55], and the resulting neutral monosaccharides reduced, acetylated and analysed by capillary gas chromatography. Total uronic acids were measured by the *m*-hydroxydiphenyl method [56] with D-galacturonic acid as the standard. All determinations were done in triplicate.

### Degree of methyl esterification (DM) of pectic polysaccharides

The DM of pectic polysaccharides was determined as described by McFeeters and Armstrong [57] with modifications. Briefly, freeze-dried cell walls (~ 3 mg) were saponified overnight at 4 °C in a mixture of 50 mM citric acid containing 1 M sodium chloride (25 μL), water (225 μL) and 1 M sodium hydroxide (20 μL). The slurry was neutralized with 82.5 mM citric acid (30 μL) followed by the addition of 25 mM *n*-propanol (33.3 μL) as the internal standard. After centrifuging (20,000×g, 5 min), an aliquot (1 μL) of the supernatant was analysed by capillary gas chromatography on a SGE™ BP20 column (15 m long, 0.32 mm i.d, 0.25 μm film thickness) (ThermoFisher Scientific, Waltham, MA, USA). The DM was calculated as methanol (mol)/ UA (mol) × 100%. Duplicate samples were analysed.

### Indirect immunofluorescence detection of cell-wall polysaccharides

This was done essentially as described by Zhang et al. [58]. Transverse segments (1–2 mm long) were cut midway along petioles at the four different stages of development, and fixed in 100 mM sodium PIPES (1,4-Piperazinediethanesulfonic acid) buffer (pH 7.2) containing 2% (w/v) paraformaldehyde and 0.1% (w/v) glutaraldehyde for 2 h at room temperature under vacuum. After washing with buffer only, the segments were dehydrated using an aqueous ethanol series (30, 50, 70, 85, 90, and 100% twice) for 15 mins at each concentration. They were then infiltrated for 1 h at room temperature with a 2:1 (v/v) mixture of ethanol and LR White resin (medium grade) (London Resin Co. Ltd., Basingstoke, UK) followed by a 1:2 (v/v) mixture of ethanol and resin for 1 h, and finally in pure resin for 18 h. They were then placed in gelatine capsules containing fresh resin and polymerized at 60 °C for 24 h.

Transverse sections (200 nm thick) were cut using a diamond knife on an ultramicrotome (see above), transferred to poly-L-lysine coated slides (Biolab Scientific, Auckland), and dried at 55 °C for 30 min. Non-specific binding sites were blocked by incubating sections in 10 mM sodium phosphate buffer (pH 7.4) containing 140 mM NaCl (PBS) and 5% (w/v) milk powder (MP-PBS) for 1 h at room temperature. The sections were then washed with PBS (4x) with shaking at 70 rpm, followed by incubation with the monoclonal antibodies (20 μL) (PlantProbes, Leeds, UK) in MP-PBS for 2 h. The following monoclonal antibodies were used: LM5, specific for (1 → 4)-β-d-galactans [18]; LM6, specific for (1 → 5)-α-L-arabinans [59]; LM19, specific for non- or low methyl esterified HG [60]; LM20, specific for more highly methyl esterified HG [60]; LM10, specific for unsubstituted or low-substituted (1 → 4)-β-d-xylans [61]; LM11, specific for more highly substituted (1 → 4)-β-d-xylans [61]; LM15, specific for xyloglucans [19]; LM21, specific for heteromannans [62]. LM19 and LM20 were diluted 1:5 (v/v) in MP-PBS before use, and other primary antibodies were diluted 1:10 (v/v) in MP-PBS. After washing with shaking in PBS (5x), the sections were incubated with the secondary antibody goat anti-rat (H + L) conjugated to Alexa Fluor® 546 (ThermoFisher Scientific) (20 μL, 1:200 dilution) in MP-PBS at room temperature for 1.5 h in the dark. After washing (with shaking) with PBS (5x), the sections were stained with 0.03% (w/v) Calcofluor White (Fluorescent Brighter 28 sodium salt, Sigma-Aldrich, St. Louis, USA) for 5 min in the dark, washed (with shaking) with PBS (3x) then water (2x), mounted in AF1 antifadent (Citifluor Ltd., London) and examined with a light microscope equipped for epifluorescence microscopy (Nikon Eclipse Ni-U microscope; Nikon Instruments Inc., Melville, NY, USA). The filter sets used were as follows: for Alexa Fluor® 546 fluorescence, the TRIC filter set (excitation filter BP 530–560 nm; chromatic beam splitter 570 nm, and emission filter BP 590–650 nm); for Calcofluor White fluorescence, the DAPI filter set (excitation filter BP 325–375 nm; chromatic beam splitter 400 nm, emission filter BP435–485 nm). Control experiments were done with the monoclonal antibody omitted. For each antibody, the same exposure time was used for all developmental stages.

For some experiments, sections were treated in one of the following two ways before non-specific binding sites were blocked: with 0.1 M Na_2_CO_3_ (pH 11.4) for 2 h at room temperature to remove methyl esters from HG; with 0.1 M Na_2_CO_3_ for 2 h, followed by pectate lyase (10 μg/mL) (from *Cellvibrio japonicus*, Megazyme International, Bray, Ireland) in 50 mM N-cyclohexyl-3-aminopropanesulfonic acid (CAPS) buffer (pH 10) containing 2 mM CaCl_2_ for 2 h at room temperature to remove HG [19]. Control experiments were also done in which sections were treated with 0.1 M Na_2_CO_3_ followed by CAPS buffer containing CaCl_2,_ but no pectate lyase.

### Indirect immunogold microscopy detection of cell-wall polysaccharides

This was done essentially as described by Zhang et al. [58]. Transverse sections (100 nm thick) were collected on 200 mesh nickel grids (ProSciTech), and some of these were pre-treated to remove methyl esters from HG or to remove HG as described for immunofluorescence microscopy. The sections were blocked, washed with shaking, and incubated with monoclonal antibodies as described for immunofluorescence microscopy, but at 4 °C overnight before washing (with shaking) with PBS (5x). The sections were then incubated with the secondary antibody goat anti-rat IgG (H + L) conjugated to 15 nm diameter colloidal gold particles (Electron Microscopy Sciences, Hatfield, PA, USA) (diluted 1:10 in MP-PBS) for 2 h at room temperature, washed (with shaking) with PBS (5x) and water (2x). The sections were then stained with 2% (w/v) aqueous uranyl acetate for 20 mins, washed with water (6x), dried and examined by TEM as described above. Control experiments were done with monoclonal antibodies omitted.

### Solid-state ^13^C NMR spectroscopy

CP/MAS solid-state NMR spectroscopy was carried out as described by Bootten et al. [24]. Freeze-dried CWs were rehydrated to a water content of 65% (w/w) using 80% (v/v) ethanol, packed in 4-mm diameter zirconia rotors and retained with Kel-F end-caps. The samples were spun at 4 kHz in a Bruker magic-angle spinning double-tuned probe for ^13^C NMR spectroscopy at 75 MHz using a Bruker Avance III 500 MHz spectrometer. For CP/MAS experiments, the 90° proton preparation pulse was 4.2 μs followed by a 1 ms CP contact time, 51 ms of data acquisition, and a recovery delay of 1 s before the sequence was repeated. A total of 12,000 transients were used.

The relaxation experiments were conducted with 4 kHz MAS at room temperature. Proton T_1ρ_ was carried out according to Hediger et al. [9] using a 400 μs contact time and 12,000 scans for each time delay. Carbon T_1_ was measured as described by Torchia [63]. The relaxation delay was set at 20 μs, 10 ms, 100 ms, 300 ms, 500 ms, 1 s, 2.5 s, 5 s, 10 s and 20 s. 3800 accumulations were used for each time delay. The decay pattern between relative peak intensities and relaxation times were fitted with mono- or bi-exponential functions in OriginPro 8 (OriginLab, Northampton, MA, USA).

## Additional files

Additional file 1: **Figure S1.** Control immunofluorescence micrographs of transverse sections of celery collenchyma strands at four developmental stages treated with Na_2_CO_3_ or Na_2_CO_3_ and CAPS buffer followed by the primary antibodies LM20 (Na_2_CO_3_), LM10, LM11 and LM21 (Na_2_CO_3_ and CAPS buffer). (DOCX 331 kb)

Additional file 2: **Figure S2.** Control immunofluorescence micrographs of transverse sections of celery collenchyma strands at four developmental stages with the omission of the primary antibodies LM19, LM20, LM5 and LM6. (DOCX 169 kb)

Additional file 3: **Figure S3.** Control immunofluorescence micrographs of transverse sections of celery collenchyma strands at four developmental stages with the omission of the primary antibodies LM15, LM10, LM11 and LM21. (DOCX 261 kb)

Additional file 4: **Figure S4.** Immunogold labelling patterns of thin regions of celery collenchyma cell walls at four developmental stages with LM19, LM20, LM5, LM6 and LM15. (DOCX 1130 kb)

Additional file 5: **Figure S5.** Control immunogold micrographs of transverse sections of celery collenchyma strands at four developmental stages with the omission of the primary antibodies. (DOCX 867 kb)

Additional file 6: **Figure S6.** Control immunogold micrographs of transverse sections of celery collenchyma strands at four developmental stages pre-treated with pectate lyase with the omission of the primary antibodies. (DOCX 883 kb)

Additional file 7: **Figure S7.** CP/MAS NMR relaxation spectra of celery collenchyma cell walls at developmental stage 4 obtained using various delay times. (DOCX 58 kb)

## Abbreviations

CAPS: N-cyclohexyl-3-aminopropanesulfonic acid
CP/MAS NMR: cross polarization with magic angle spinning nuclear magnetic resonance
HEPES: 4-(2-Hydroxyethyl)piperazine-1-ethanesulfonic acid
HG: homogalacturonan
PBS: 10 mM sodium phosphate buffer (pH 7.4) containing 140 mM NaCl
PIPES: 1,4-Piperazinediethanesulfonic acid
PME: pectin methylesterases
RG-I: rhamnogalacturonan I
RG-II: rhamnogalacturonan II
T_1_^C^: spin-lattice relaxation time constant of ^13^C
T_1ρ_^H^: rotating-frame relaxation time constant of protons
TFA: trifluoroacetic acid

## References

[1] Fahn A (1967). Plant Anatomy.

[2] Evert RF. Esau’s plant anatomy: meristems, cells, and tissues of the plant body-their structure, function, and development. 3rd ed. New Jersey: Wiley; 2006. p. 183–90.

[3] Leroux O (2012). Collenchyma: a versatile mechanical tissue with dynamic cell walls. Ann Bot.

[4] Chen D, Harris PJ, Sims IM, Zujovic Z, Melton LD (2017). Polysaccharide compositions of collenchyma cell walls from celery (*Apium graveolens* L.) petioles. BMC Plant Biol.

[5] Majumdar GP, Preston RD (1941). The fine structure of collenchyma cells in *Heracleum sphondylium* L. Proc R Soc Lond B.

[6] Vian B, Roland JC, Reis D (1993). Primary cell wall texture and its relation to surface expansion. Int J Plant Sci.

[7] Thimm JC, Burritt DJ, Sims IM, Newman RH, Ducker WA, Melton LD (2002). Celery (*Apium graveolens*) parenchyma cell walls: cell walls with minimal xyloglucan. Physiol Plant.

[8] Zujovic Z, Chen D, Melton LD (2016). Comparison of celery (*Apium graveolens* L.) collenchyma and parenchyma cell wall polysaccharides enabled by solid-state ^13^C NMR. Carbohydr Res.

[9] Hediger S, Emsley L, Fischer M (1999). Solid-state NMR characterization of hydration effects on polymer mobility in onion cell-wall material. Carbohydr Res.

[10] Jarvis MC, Apperley DC (1990). Direct observation of cell wall structure in living plant tissues by solid-state ^13^C NMR spectroscopy. Plant Physiol.

[11] Fenwick KM, Jarvis MC, Apperley DC. Estimation of polymer rigidity in cell walls of growing and nongrowing celery collenchyma by solid-state nuclear magnetic resonance *in vivo*. Plant Physiol. 1997;115:587–92. 10.1104/pp.115.2.587 PMC158518 12223826

[12] Thomas LH, Forsyth VT, Šturcová A, Kennedy CJ, May RP, Altaner CM, Apperley DC, Wess TJ, Jarvis MC (2013). Structure of cellulose microfibrils in primary cell walls from collenchyma. Plant Physiol.

[13] Chafe SC (1970). The fine structure of the collenchyma cell wall. Planta.

[14] Reeve RM (1958). A specific hydroxylamine-ferric chloride reaction for histochemical localization of pectin. Stain Technol.

[15] Albersheim P, Mühlethaler K, Frey-Wyssling A (1960). Stained pectin as seen in the electron microscope. J Biophys Biochem Cytol.

[16] Ng JK, Schröder R, Sutherland PW, Hallett IC, Hall MI, Prakash R, Smith BG, Melton LD, Johnston JW (2013). Cell wall structures leading to cultivar differences in softening rates develop early during apple (*Malus x domestica*) fruit growth. BMC Plant Biol.

[17] Lee KJ, Knox JP, Žárský V, Cvrčková F (2014). Resin embedding, sectioning, and immunocytochemical analyses of plant cell walls in hard tissues. Plant cell morphogenesis. Methods in molecular biology (methods and protocols).

[18] Jones L, Seymour GB, Knox JP (1997). Localization of pectic galactan in tomato cell walls using a monoclonal antibody specific to (1→4)-β-D-galactan. Plant Physiol.

[19] Marcus SE, Verhertbruggen Y, Hervé C, Ordaz-Ortiz JJ, Farkas V, Pedersen HL, Willats WG, Knox JP (2008). Pectic homogalacturonan masks abundant sets of xyloglucan epitopes in plant cell walls. BMC Plant Biol.

[20] Hervé C, Rogowski A, Gilbert HJ, Knox PJ (2009). Enzymatic treatments reveal differential capacities for xylan recognition and degradation in primary and secondary plant cell walls. Plant J.

[21] Esau K (1936). Ontogeny and structure of collenchyma and of vascular tissues in celery petioles. Hilgardia.

[22] Beer M, Setterfield G (1958). Fine structure in thickened primary walls of collenchyma cells of celery petioles. Am J Bot.

[23] Bootten TJ, Harris PJ, Melton LD, Newman RH (2004). Solid-state ^13^C-NMR spectroscopy shows that the xyloglucans in the primary cell walls of mung bean (*Vigna radiata* L.) occur in different domains: a new model for xyloglucan–cellulose interactions in the cell wall. J Exp Bot.

[24] Bootten TJ, Harris PJ, Melton LD, Newman RH, Popper ZA (2011). Using solid-state ^13^C NMR spectroscopy to study the molecular organisation of primary plant cell walls. The plant cell wall: methods and protocols.

[25] White PB, Wang T, Park YB, Cosgrove DJ, Hong M (2014). Water–polysaccharide interactions in the primary cell wall of *Arabidopsis thaliana* from polarization transfer solid-state NMR. J Am Chem Soc.

[26] Derbyshire P, McCann MC, Roberts K (2007). Restricted cell elongation in *Arabidopsis* hypocotyls is associated with a reduced average pectin esterification level. BMC Plant Biol.

[27] Hall HC, Cheung J, Ellis BE (2013). Immunoprofiling reveals unique cell-specific patterns of wall epitopes in the expanding *Arabidopsis* stem. Plant J.

[28] Grant GT, Morris ER, Rees DA, Smith PJ, Thom D (1973). Biological interactions between polysaccharides and divalent cations: the egg-box model. FEBS Lett.

[29] Parre E, Geitmann A (2005). Pectin and the role of the physical properties of the cell wall in pollen tube growth of *Solanum chacoense*. Planta.

[30] Levesque-Tremblay G, Pelloux J, Braybrook SA (2015). Tuning of pectin methylesterification: consequences for cell wall biomechanics and development. Planta.

[31] Mohnen D (2008). Pectin structure and biosynthesis. Curr Opin Plant Biol.

[32] Willats WG, Steele-King CG, Marcus SE, Knox JP (1999). Side chains of pectic polysaccharides are regulated in relation to cell proliferation and cell differentiation. Plant J.

[33] Verhertbruggen Y, Marcus SE, Haeger A, Verhoef R, Schols HA, McCleary BV, McKee L, Gilbert HJ, Paul Knox J (2009). Developmental complexity of arabinan polysaccharides and their processing in plant cell walls. Plant J.

[34] Bush MS, Marry M, Huxham MI, Jarvis MC, McCann MC (2001). Developmental regulation of pectic epitopes during potato tuberisation. Planta.

[35] Ulvskov P, Wium H, Bruce D, Jørgensen B, Qvist KB, Skjøt M (2005). Biophysical consequences of remodeling the neutral side chains of rhamnogalacturonan I in tubers of transgenic potatoes. Planta.

[36] Zykwinska A, Ralet MC, Garnier CD, Thibault J-F. Evidence for *in vitro* binding of pectin side chains to cellulose. Plant Physiol. 2005;139:397–407. 10.1104/pp.105.065912 PMC1203388 16126855

[37] Hwang J, Kokini JL (1991). Structure and rheological function of side branches of carbohydrate polymers. J Texture Stud.

[38] Jones L, Milne JL, Ashford D, McQueen-Mason SJ (2003). Cell wall arabinan is essential for guard cell function. Pro Natl Acad Sci USA.

[39] Moore JP, Farrant JM, Driouich A (2008). A role for pectin-associated arabinans in maintaining the flexibility of the plant cell wall during water deficit stress. Plant Signal Behav.

[40] Moore JP, Nguema-Ona EE, Vicré-Gibouin M, Sørensen I, Willats WG, Driouich A, Farrant JM (2013). Arabinose-rich polymers as an evolutionary strategy to plasticize resurrection plant cell walls against desiccation. Planta.

[41] Daher FB, Braybrook SA (2015). How to let go: pectin and plant cell adhesion. Front Plant Sci.

[42] Ordaz-Ortiz JJ, Marcus SE, Knox JP (2009). Cell wall microstructure analysis implicates hemicellulose polysaccharides in cell adhesion in tomato fruit pericarp parenchyma. Mol Plant.

[43] Guillon F, Moïse A, Quemener B, Bouchet B, Devaux MF, Alvarado C, Lahaye M (2017). Remodeling of pectin and hemicelluloses in tomato pericarp during fruit growth. Plant Sci.

[44] Hayashi T, Kaida R (2011). Functions of xyloglucan in plant cells. Mol Plant.

[45] Popper ZA, Fry SC (2008). Xyloglucan-pectin linkages are formed intra-protoplasmically, contribute to wall-assembly, and remain stable in the cell wall. Planta.

[46] Scheller HV, Ulvskov P (2010). Hemicelluloses. Plant Biol.

[47] Bidhendi AJ, Geitmann A (2016). Relating the mechanics of the primary plant cell wall to morphogenesis. J Exp Bot.

[48] Vincken JP, Schols HA, Oomen RJ, McCann MC, Ulvskov P, Voragen AG, Visser RG (2003). If homogalacturonan were a side chain of rhamnogalacturonan I. Implications for cell wall architecture. Plant Physiol.

[49] Wang T, Yang H, Kubicki JD, Hong M (2016). Cellulose structural polymorphism in plant primary cell walls investigated by high-field 2D solid-state NMR spectroscopy and density functional theory calculations. Biomacromolecules.

[50] Wang T, Zabotina O, Hong M (2012). Pectin-cellulose interactions in the *Arabidopsis* primary cell wall from two-dimensional magic-angle-spinning solid-state nuclear magnetic resonance. Biochemistry.

[51] Reynolds ES (1963). The use of lead citrate at high pH as an electron-opaque stain in electron microscopy. J Cell Biol.

[52] Melton LD, Smith BG. Isolation of plant cell walls and fractionation of cell wall polysaccharides. In: Wrolstad RE, Acree TE, editors. Handbook of food analytical chemistry: water, proteins, enzymes, lipids and carbohydrates. New Jersey. USA: Wiley & Sons; 2005. p. 697–719.

[53] Albersheim P, Nevins DJ, English PD, Karr A (1967). A method for the analysis of sugars in plant cell-wall polysaccharides by gas-liquid chromatography. Carbohydr Res.

[54] Harris PJ, Blakeney AB, Henry RJ, Stone BA. Gas-chromatographic determination of the monosaccharide composition of plant cell wall preparations. J Assoc Off Anal Chem. 1988;71:272–5.

[55] Saeman JF, Moore WE, Millet MA, Whistler RL (1963). Sugar units present. Hydrolysis and quantitative paper chromatography. Methods in carbohydrate chemistry.

[56] Filisetti-Cozzi TMCC, Carpita NC (1991). Measurement of uronic acids without interference from neutral sugars. Anal Biochem.

[57] McFeeters RF, Armstrong SA (1984). Measurement of pectin methylation in plant cell walls. Anal Biochem.

[58] Zhang M, Chavan RR, Smith BG, McArdle BH, Harris PJ (2017). Tracheid cell-wall structures and locations of (1→4)-β-D-galactans and (1→3)-β-D-glucans in compression woods of radiata pine (*Pinus radiata* D. Don). BMC Plant Biol.

[59] Willats WG, Marcus SE, Knox JP (1998). Generation of a monoclonal antibody specific to (1→5)-*α*-L-arabinan. Carbohydr Res.

[60] Verhertbruggen Y, Marcus SE, Haeger A, Ordaz-Ortiz JJ, Knox JP (2009). An extended set of monoclonal antibodies to pectic homogalacturonan. Carbohydr Res.

[61] McCartney L, Marcus SE, Knox JP (2005). Monoclonal antibodies to plant cell wall xylans and arabinoxylans. J Histochem Cytochem.

[62] Marcus SE, Blake AW, Benians TA, Lee KJ, Poyser C, Donaldson L, Leroux O, Rogowski A, Petersen HL, Boraston A, Gilbert HJ (2010). Restricted access of proteins to mannan polysaccharides in intact plant cell walls. Plant J.

[63] Torchia DA (1978). The measurement of proton-enhanced carbon-13 T_1_ values by a method which suppresses artifacts. J Magn Reson.

