# Supplementary material for: Developmental changes in collenchyma cell-wall polysaccharides in celery (Apium graveolens L.) petioles
Source: BMC Plant Biol. 2019 Feb 19;19:81. doi: 10.1186/s12870-019-1648-7 (PMC6381709; doi:10.1186/s12870-019-1648-7)
Supplement: Supplementary file 1 — Figure S1. Control immunofluorescence micrographs of transverse sections of celery collenchyma strands at four developmental stages treated with Na2CO3 or Na2CO3 and CAPS buffer followed by the primary antibodies LM20 (Na2CO3), LM10, LM11 and LM21 (Na2CO3 and CAPS buffer). (DOCX 331 kb) [file 12870_2019_1648_MOESM1_ESM.docx]

**Additional file 1**


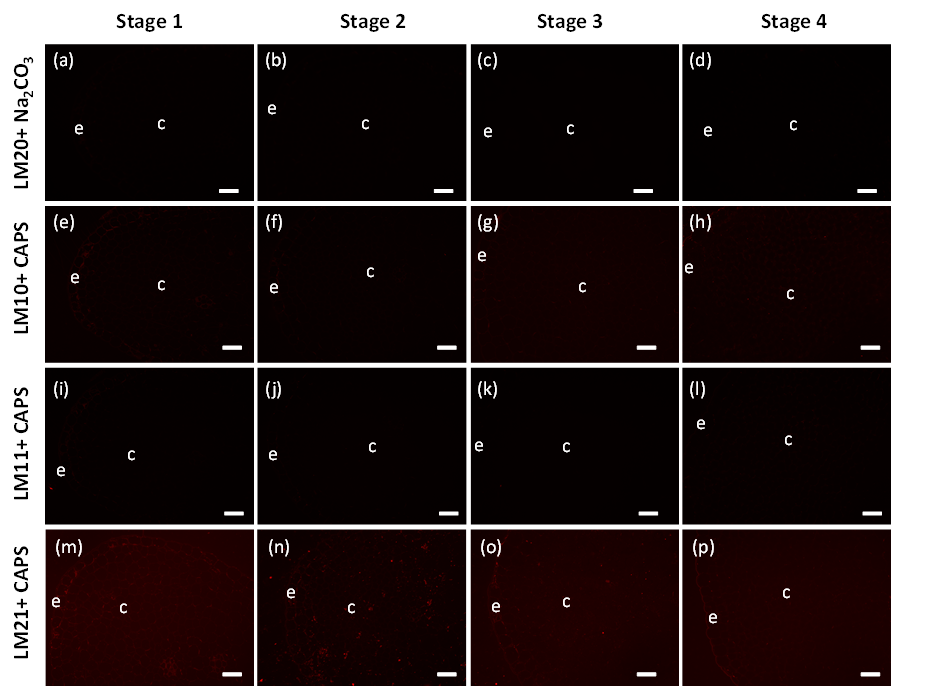


**Figure S1.** Control immunofluorescence micrographs of transverse sections of celery collenchyma strands at four developmental stages pretreated with Na_2_CO_3_ or Na_2_CO_3_  and CAPS buffer followed by labelling with the primary antibodies LM20 (**a**-**d**) (Na_2_CO_3_), LM10 (**e**-**h**), LM11 (**i**-**l**) and LM21 (**m**-**p**) (Na_2_CO_3_ and CAPS buffer). LM20+ Na_2_CO_3_, sections were pretreated with sodium carbonate before labelling with LM20. LM10+ CAPS, LM11+ CAPS, LM21+ CAPS are control experiments in which sections were pretreated with sodium carbonate followed by 50 mM CAPS buffer containing 2 mM CaCl_2_ (pH10), but no pectate lyase, before labelling with LM10, LM11 and LM21. Stage 1 (from 2.6 cm long petiole) (**a**, **e**, **i**, **m**); Stage 2 (from 11 cm petiole) (**b**, **f**, **j**, **n**); Stage 3 (from 24 cm petiole) (**c**, **g**, **k**, **o**); Stage 4 (from 40 cm petiole) (**d**, **h**, **l**, **p**). e = epidermis, c = collenchyma cells. Scale= 50 μm.
