# Supplementary material for: Developmental changes in collenchyma cell-wall polysaccharides in celery (Apium graveolens L.) petioles
Source: BMC Plant Biol. 2019 Feb 19;19:81. doi: 10.1186/s12870-019-1648-7 (PMC6381709; doi:10.1186/s12870-019-1648-7)
Supplement: Supplementary file 2 — Figure S2. Control immunofluorescence micrographs of transverse sections of celery collenchyma strands at four developmental stages with the omission of the primary antibodies LM19, LM20, LM5 and LM6. (DOCX 169 kb) [file 12870_2019_1648_MOESM2_ESM.docx]

**Additional file 2**


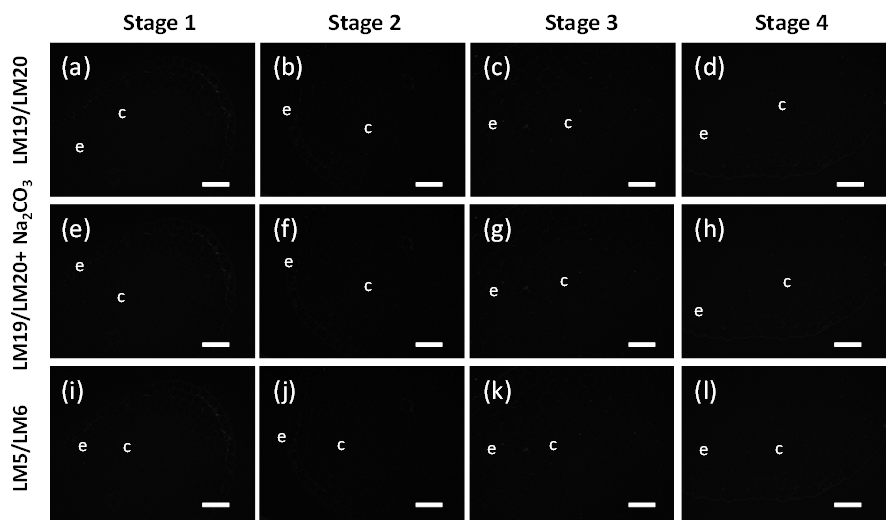


**Figure S2.** Control immunofluorescence micrographs of transverse sections of celery collenchyma strands at four developmental stages with the omission of the primary antibodies LM19, LM20, LM5 and LM6. LM19/ LM20 (**a**-**d**) are control experiments for sections with LM19 or LM20 omitted. LM19/LM20 + Na_2_CO_3_ (**e**-**h**) are control experiment for sections pretreated with Na_2_CO_3_, but with LM19 or LM20 omitted. LM5/LM6 (**i-l**) are control experiments for sections with LM5 or LM6 omitted. Stage 1 (from 2.6 cm petiole) (**a**, **e**, **i**); Stage 2 (from 11 cm petiole) (**b**, **f**, **j**); Stage 3 (from 24 cm petiole) (**c**, **g**, **k**); Stage 4 (from 40 cm petiole) (**d**, **h**, **l**). e = epidermis, c = collenchyma cells. Scale = 100 μm.
