# Supplementary material for: Developmental changes in collenchyma cell-wall polysaccharides in celery (Apium graveolens L.) petioles
Source: BMC Plant Biol. 2019 Feb 19;19:81. doi: 10.1186/s12870-019-1648-7 (PMC6381709; doi:10.1186/s12870-019-1648-7)
Supplement: Supplementary file 3 — Figure S3. Control immunofluorescence micrographs of transverse sections of celery collenchyma strands at four developmental stages with the omission of the primary antibodies LM15, LM10, LM11 and LM21. (DOCX 261 kb) [file 12870_2019_1648_MOESM3_ESM.docx]

**Additional file 3**


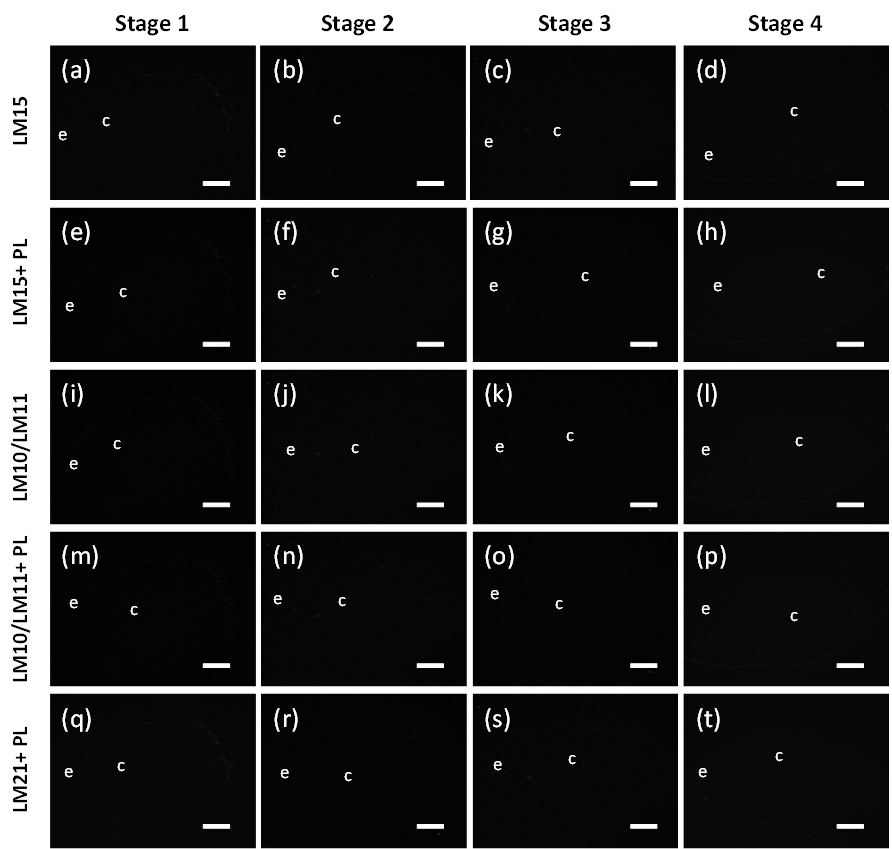


**Figure S3.** Control immunofluorescence micrographs of transverse sections of celery collenchyma strands at four developmental stages with the omission of the primary antibodies LM15, LM10, LM11 and LM21. LM15 (**a**-**d**) are control experiments for sections with LM15 labelling omitted. LM15 + PL (**e**-**h**) are control experiments for sections pretreated with pectate lyase, but with LM15 labelling omitted. LM10/LM11 (**i-l**) are control experiments for sections with LM10 or LM11 labelling omitted. LM10/LM11+ PL (**m-p**) are control experiments for sections pretreated with pectate lyase, but with LM10 or LM11 labelling omitted. LM21+ PL (**q-t**) are control experiments for sections pretreated with pectate lyase, but with LM21 labelling omitted. Stage 1 (from 2.6 cm petiole) (**a**, **e**, **I, m, q**); Stage 2 (from 11 cm petiole) (**b**, **f**, **j, n, r**); Stage 3 (from 24 cm petiole) (**c**, **g**, **k, o, s**); Stage 4 (from 40 cm petiole) (**d**, **h**, **l, p, t**). e = epidermis, c = collenchyma cells. Scale = 100 μm.
