# Supplementary material for: Developmental changes in collenchyma cell-wall polysaccharides in celery (Apium graveolens L.) petioles
Source: BMC Plant Biol. 2019 Feb 19;19:81. doi: 10.1186/s12870-019-1648-7 (PMC6381709; doi:10.1186/s12870-019-1648-7)
Supplement: Supplementary file 4 — Figure S4. Immunogold labelling patterns of thin regions of celery collenchyma cell walls at four developmental stages with LM19, LM20, LM5, LM6 and LM15. (DOCX 1130 kb) [file 12870_2019_1648_MOESM4_ESM.docx]

**Additional file 4**


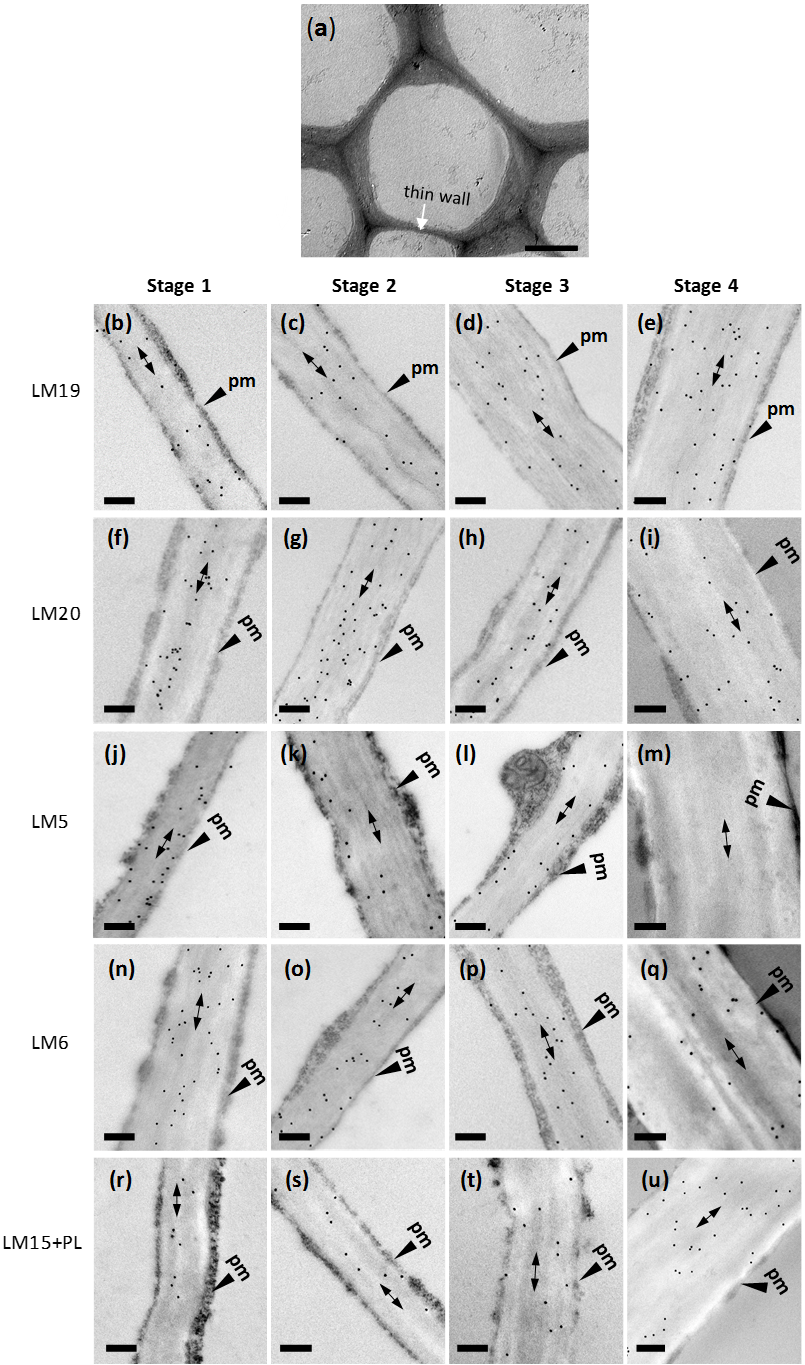


**Figure S4.** Immunogold labelling of thin regions of celery collenchyma cell walls at four stages of development using LM19 (**b-e**), LM20 (**f-i**), LM5 (**j-m**), LM6 (**n-q**) and LM15 (**r-u**). LM15+PL, the transverse sections were pretreated with pectate lyase before labelling with LM15. Stage 1 (**b, f, j, n, r**), 2.6 cm petioles; Stage 2 (**c, g, k, o, s**), 11 cm petioles; Stage 3 (**d, h, l, p, t**), -24 cm petioles; Stage 4 (**e, i, m, q, u**), -40 cm petioles (**a**) A TEM micrograph of transverse section of a collenchyma cell at Stage 4 (39 cm petiole); the thin cell-wall region is shown with an arrow. Scale bar = 5 μm. (**b-u**) Double head arrows show the location of middle lamella. pm, plasma membrane. Scale = 200 nm.
