# Supplementary material for: Developmental changes in collenchyma cell-wall polysaccharides in celery (Apium graveolens L.) petioles
Source: BMC Plant Biol. 2019 Feb 19;19:81. doi: 10.1186/s12870-019-1648-7 (PMC6381709; doi:10.1186/s12870-019-1648-7)
Supplement: Supplementary file 6 — Figure S6. Control immunogold micrographs of transverse sections of celery collenchyma strands at four developmental stages pre-treated with pectate lyase with the omission of the primary antibodies. (DOCX 883 kb) [file 12870_2019_1648_MOESM6_ESM.docx]

**Additional file 6**


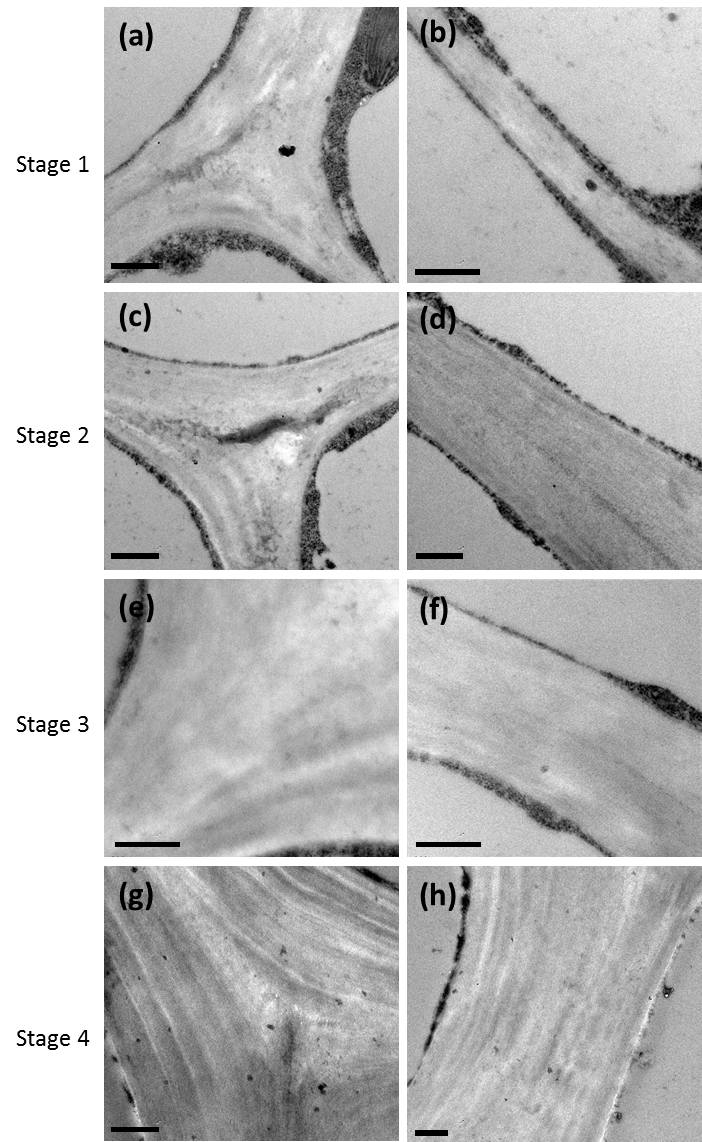


**Figure S6**. Control images of immunogold labelling of pectate lyase pre-treated transverse section of celery collenchyma strands at four developmental stages with the omission of the primary antibodies. Stage 1 (**a**, **b**), Stage 2 (**c**, **d**), Stage 3 (**e**, **f**), Stage 4 (**g**, **h**). Scale bars= 500 nm.
