# Supplementary material for: Developmental changes in collenchyma cell-wall polysaccharides in celery (Apium graveolens L.) petioles
Source: BMC Plant Biol. 2019 Feb 19;19:81. doi: 10.1186/s12870-019-1648-7 (PMC6381709; doi:10.1186/s12870-019-1648-7)
Supplement: Supplementary file 7 — Figure S7. CP/MAS NMR relaxation spectra of celery collenchyma cell walls at developmental stage 4 obtained using various delay times. (DOCX 58 kb) [file 12870_2019_1648_MOESM7_ESM.docx]

**Additional file 7**

**
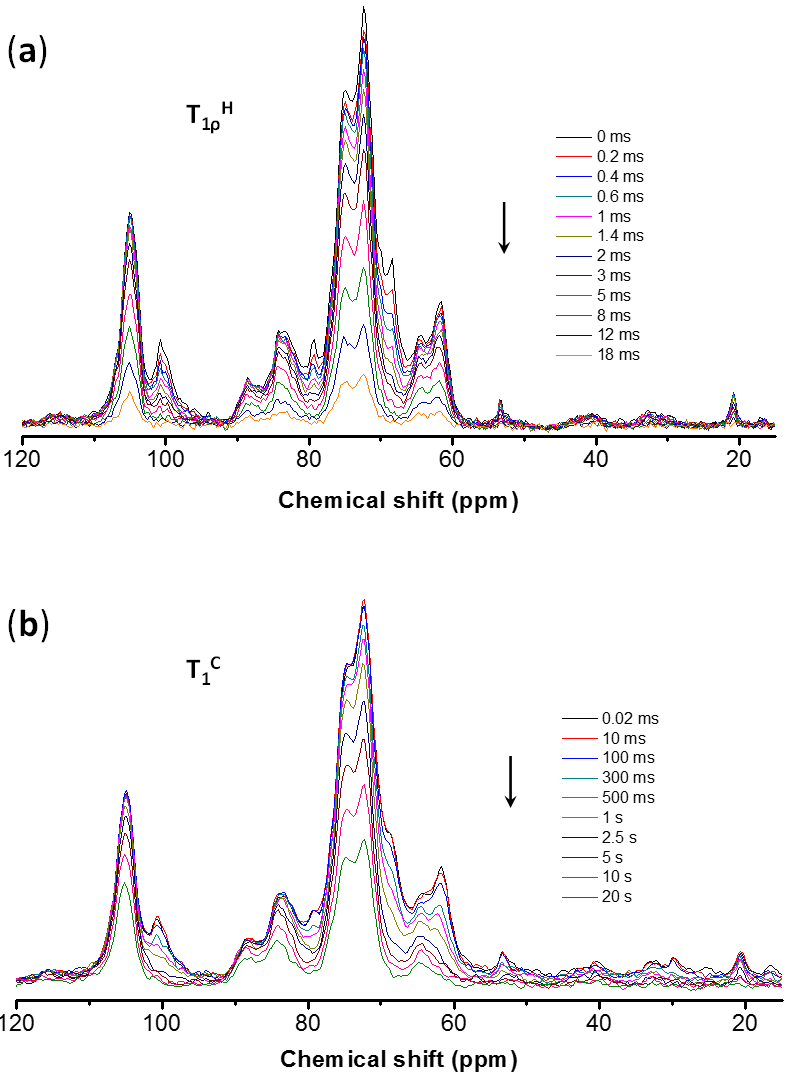
**

**Figure S7.** T_1ρ_^H^ (**a**) and T_1_^C^ (**b**) CP/MAS NMR relaxation spectra of celery collenchyma cell walls at developmental stage 4 (35-40 cm petioles) obtained using various delay times. The measurements were done at room temperature with a 65% hydration level. The peak intensities decrease gradually with increasing delay time.
